# New chromosome-scale genomes provide insights into marine adaptations of sea snakes (*Hydrophis*: Elapidae)

**DOI:** 10.1186/s12915-023-01772-2

**Published:** 2023-12-08

**Authors:** Alastair J. Ludington, Jillian M. Hammond, James Breen, Ira W. Deveson, Kate L. Sanders

**Affiliations:** 1 School of Biological Sciences, The University of Adelaide, Adelaide, SA 5005 Australia; 2 Genomics and Inherited Disease Program, Garvan Institute of Medical Research, Sydney, NSW Australia; 3 Centre for Population Genomics, Garvan Institute of Medical Research and Murdoch Children’s Research Institute, Darlinghurst, Australia; 4 Indigenous Genomics, Telethon Kids Institute, Adelaide, Australia; 5 John Curtin School of Medical Research, College of Health & Medicine, Australian National University, Canberra, Australia; 6 Faculty of Medicine, University of New South Wales, Sydney, NSW Australia; 7 The South Australian Museum, Adelaide, Australia

**Keywords:** Chromosome-scale genome, Synteny, Positive selection, Marine adaptation, Sea snake

## Abstract

**Background:**

Sea snakes underwent a complete transition from land to sea within the last ~ 15 million years, yet they remain a conspicuous gap in molecular studies of marine adaptation in vertebrates.

**Results:**

Here, we generate four new annotated sea snake genomes, three of these at chromosome-scale (*Hydrophis major*, *H*. *ornatus* and *H. curtus*), and perform detailed comparative genomic analyses of sea snakes and their closest terrestrial relatives. Phylogenomic analyses highlight the possibility of near-simultaneous speciation at the root of *Hydrophis*, and synteny maps show intra-chromosomal variations that will be important targets for future adaptation and speciation genomic studies of this system. We then used a strict screen for positive selection in sea snakes (against a background of seven terrestrial snake genomes) to identify genes over-represented in hypoxia adaptation, sensory perception, immune response and morphological development.

**Conclusions:**

We provide the best reference genomes currently available for the prolific and medically important elapid snake radiation. Our analyses highlight the phylogenetic complexity and conserved genome structure within *Hydrophis*. Positively selected marine-associated genes provide promising candidates for future, functional studies linking genetic signatures to the marine phenotypes of sea snakes and other vertebrates.

**Supplementary Information:**

The online version contains supplementary material available at 10.1186/s12915-023-01772-2.

## Background

Major evolutionary transitions, such as from terrestrial to marine habitats, present powerful opportunities to understand genomic mechanisms underlying adaptation. In the genomes of secondarily marine mammals and marine-diving birds, hundreds of genes linked to metabolic and cellular processes, physiology and functional morphology have been identified as under diversifying or relaxed selection pressures [1–5]. Far fewer studies have examined the specific genomic changes that have accompanied marine transitions in reptiles [6, 7] even though snakes and lizards have become important models for studying genome evolution [8].

Reptiles, mammals and birds must have encountered many of the same challenges during their marine transitions, particularly the biomechanical and energetic demands of aquatic locomotion, low levels of oxygen (hypoxia) during extended submergence, maintenance of body water balance in a hyperosmotic environment, dramatically shifted sensory perception and novel pathogenic environments. Reptiles also have special adaptations to marine life that are not found in mammalian and avian divers, and the many distinct aspects of reptile physiology, particularly ectothermy and flexible vascular circulation [9], might lead to different adaptive solutions. In these respects, marine reptiles are important taxa for advancing genomic studies of aquatic adaptations in vertebrates.

By far the most specialised and species-rich lineage of extant aquatic reptiles are the viviparous sea snakes (Elapidae: Hydrophiinae) [10]. This group comprises more than 60 known species that share a recent marine origin, having descended from Australo-Papuan terrestrial hydrophiines (taipans, death adders, tiger snakes, etc.) only ≈9–18 million years ago [6, 11, 12]. Sea snakes possess a suite of marine-associated characteristics: they have dorsoventrally elongate bodies and paddle-shaped tails that together generate propulsive thrust [13, 14], haemostatic nostrils that are sealed underwater by erectile tissue, and a sublingual salt-secreting gland. Specialised respiratory traits allow them to remain active underwater for extended periods, particularly a high degree of cutaneous gas exchange [15–17] that is facilitated by low partial pressure of oxygen in the arterial blood [18, 19]. The sensory systems of sea snakes have also diverged from those of their terrestrial counterparts: the visual pigments of many sea snakes have spectral sensitivities that are shifted towards the longer wavelengths that dominate marine environments [20], sea snake mechanosensory scale organs are often large and protruding [21, 22], and some species are able to withdraw their vulnerable tail paddle in response to light, a sense shared only with distantly related fish and aquatic amphibians [23].

To date, a lack of high-quality genomic resources for sea snakes and their closest terrestrial relatives has hindered genomic studies of the land-sea transition in elapids. To address this, we assembled and annotated four new sea snake genomes, three of these at chromosome scale. Analyses of newly generated (*Hydrophis major*, *H*. *ornatus*, *H. curtus* (West) and *H. elegans*) and existing (*H. curtus* (East) and *H. cyanocinctus*) data identified many candidate positively selected genes specific to *Hydrophis*, uncovered macro- and micro-chromosomal rearrangements among marine and terrestrial species, and highlighted the phylogenetic challenges of resolving the initial rapid radiation in *Hydrophis*.

## Results

### Genome assembly and annotation

**Fig. 1:**
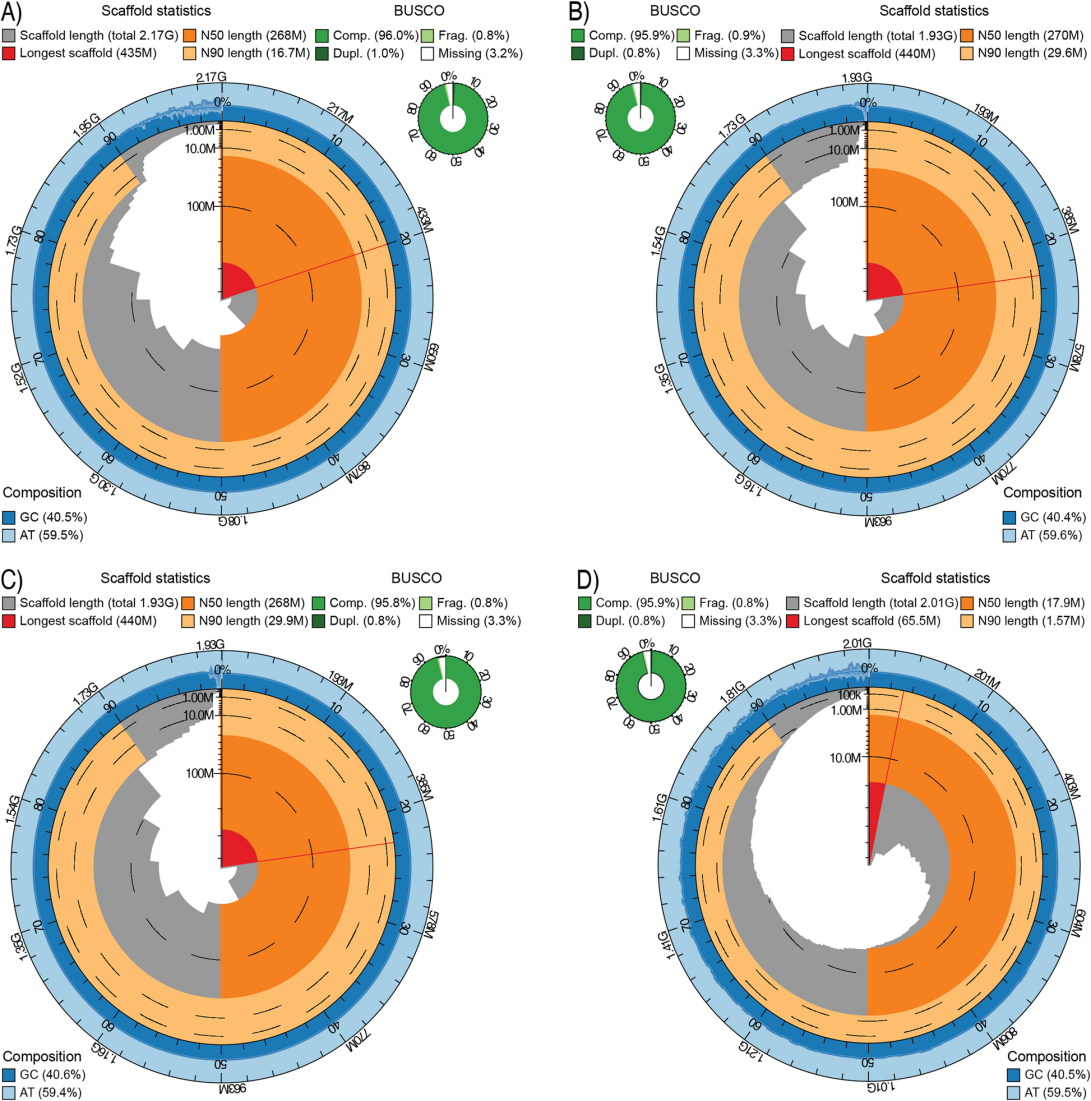
Snail plots summarising each of the *de* *novo* assembled *Hydrophis* sea snakes. For each assembly, chromosomes are arranged by length clockwise around the circle, with the largest chromosome/scaffold represented by the red segment and line at the start. The dark and light orange sections represent the N50 and N90 values, respectively, while the dark and light blue rings represent the GC and AT content in the genome. BUSCO scores for each genome are presented in the green ring next to each snail plot. Facets represent **A** *H*. *major*, **B** *H*. *ornatus*, **C** *H*. *curtus* (West) and **D** *H*. *elegans*

The differing assembly strategies implemented for *Hydrophis major*, *H*. *ornatus* and *H. curtus* (West) and *H. elegans* generated highly accurate and contiguous assemblies. The assembly process yielded a primary chromosomal assembly and dual assembly (haplotypes) for *H. major*, two chromosome-level assemblies for *H. ornatus* and *H. curtus* (West) and a highly contiguous contig assembly for *H. elegans* (Fig. 1; Table 1; Additional file 1: Figs. S1 and S2; Additional file 2: Tables S2 and S3). The karyotypes of the chromosome-scale assemblies were consistent, identifying six macro-chromosomes, nine micro-chromosomes and the Z sex chromosome (Additional file 1: Figs. S2 and S4). Numerous completeness metrics supported the accuracy and quality of the newly assembled reference genomes. The length of the assembled genomes was consistent with their estimated genome sizes (Table 1; Additional file 1: Fig. S5; Additional file 2: Table S2) and previously reported *Hydrophis* snakes [6], with chromosome sequences accounting for 90–98.7% of the total sequence length (Additional file 2: Table S2). Genome completeness was assessed using k-mer spectra analysis, with 94.9–97.3% of the sequence read k-mers being accounted for in each primary assembly (Table 1; Additional file 1: Figs. S6A-C and S7A-F; Additional file 2: Table S4), increasing to 98.9% for the *H. major* dual assembly (Additional file 1: Fig. S6D). The assembly consensus qualities (QV) surpassed 99.9% accuracy (QV30), ranging between QV34.6 and 61.5 (Table 1; Additional file 2: Table S4), while BUSCO completeness scores surpassed 95% for each assembly (Fig. 1; Table 1; Additional file 1: Fig. S8).

**Table 1:** Summary statistics for the four newly assembled *Hydrophis* genomes

	***Hydrophis major***	***Hydrophis ornatus***	***Hydrophis curtus (West)***	***Hydrophis elegans***
**Sequencing data**	HiFi + Hi-C	Nanopore + Hi-C + Short-read	Nanopore + Hi-C + Short-read	Nanopore + Short-read
**Status**	Chromosome	Chromosome	Chromosome	Contig
**Assembly size (Gbp)**				
Total	2.17	1.93	1.93	2.01
Chromosome	1.96	1.90	1.88	
Estimated	1.88	1.95	1.93	1.82
**Contig number**	1815	1399	3208	3126
**Scaffold number**	1320	548	809	3126
**Assembly N50 (Mbp)**	268	270	268	17.9
**BUSCO (complete %)**	96.0	95.9	95.8	95.9
**QV**	61.5	47.3	46.4	34.6
**K-mer completeness (%)**	95.5	97.3	95.9	94.9
**LAI**	23.9	26.2	26.7	25.9
**Gene count**	30,425	27,688	27,701	28,381
**Repeat content (% of genome)**	60.3	56.7	56.2	58.0

De novo gene prediction was performed for *H. major* and the two previously published *H. cyanocinctus* and *H. curtus* reference genomes [6], as their annotations were not available at the time of writing. *H. curtus* is currently recognised as a single widespread species, but contains two deeply divergent (species-level) lineages [24]; the Indian Ocean lineage is represented by the *H. curtus* genome generated here (referred to here as ‘*H. curtus* (West)’) and the Southeast Asian-Australian lineage is represented by the *H. curtus* genome from Li et al. [6] (‘*H. curtus* (East)’). A total of 30,425 non-redundant protein-coding genes were identified in *H. major*, 26,730 in *H. curtus* (East) and 27,689 in *H. cyanocinctus*. Lift-over annotations were generated for *H. ornatus*, *H. curtus* (West) and *H. elegans* from *H. major* as RNA-sequencing was not available for these samples, resulting in 27,688–28,381 gene predictions (Table 1; Additional file 2: Table S5). De novo gene annotations reported similar feature characteristics to RefSeq annotated snakes (Additional file 1: Fig. S9), typically reporting slightly lower average numbers of exon/coding sequences of shorter length (Additional file 2: Table S5), a phenomenon that may be explained by the excess of short gene models predicted by the de novo pipeline. BUSCO completeness measures were high for all de novo and lift-over gene annotations, surpassing 90% completeness, except for samples *H. curtus* (East) and *H. cyanocinctus*, which reached 85.4 and 85.6% completeness, respectively (Additional file 1: Fig. S10).

### Repeat annotation and genome size

**Fig. 2:**
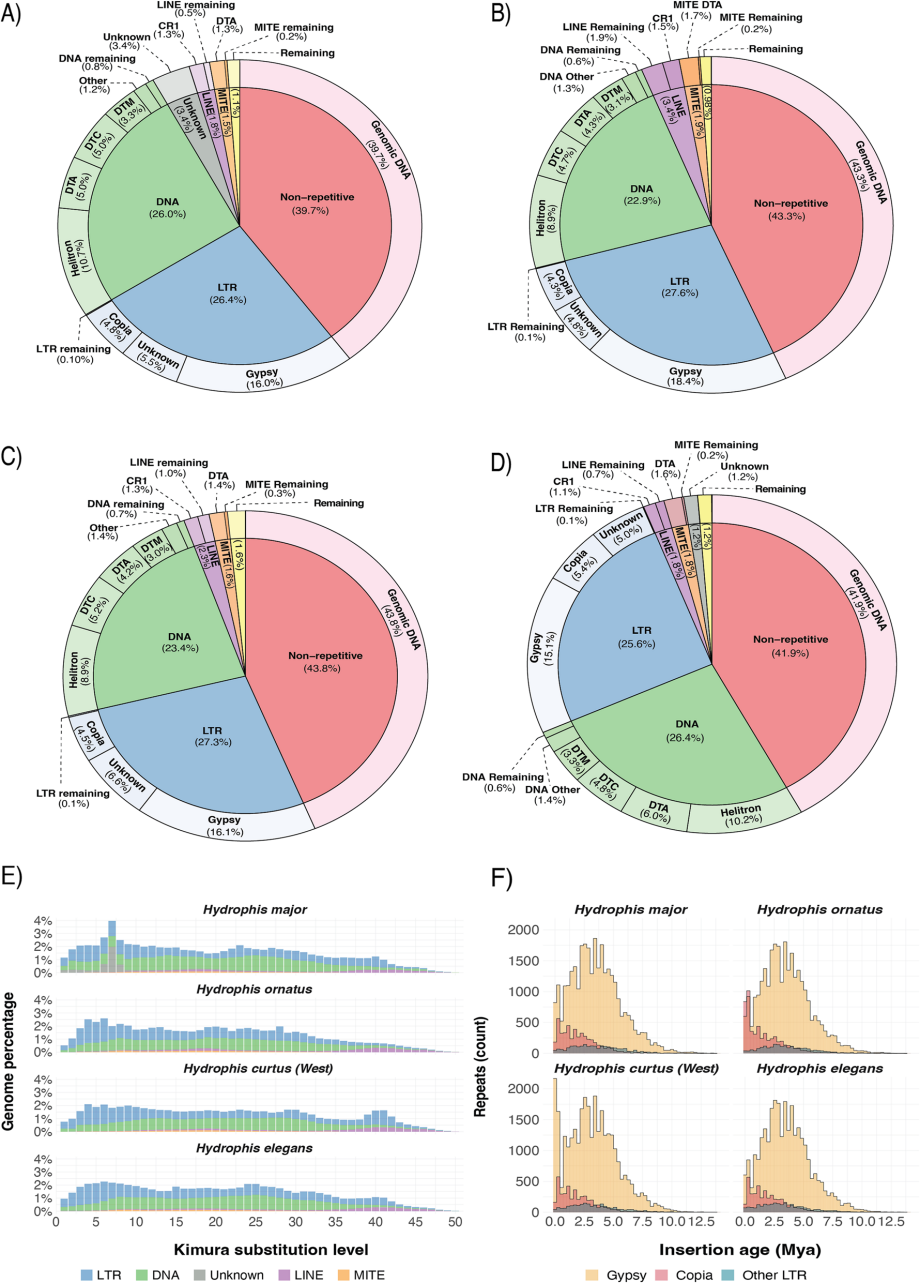
Summary of repeat annotations in the four de novo repeat-annotated sea snakes. **A-D** PieDonut repeat-summaries for the four *Hydrophis* snakes: **A** *Hydrophis major*, **B** *Hydrophis ornatus*, **C** *Hydrophis curtus* (West), **D** *Hydrophis elegans*. The inner circle represents the broad classification, with the outer donut ring consisting of the sub-family percentages. **E** Distribution of sequence divergence between TEs in each of the four *Hydrophis* assemblies relative to consensus references. The *x*-axis is the Kimura 2-parameter sequence divergence estimate, while the *y*-axis is the percentage of each genome that is annotated as TEs. **F** Insertion ages of LTR elements in the *Hydrophis* snakes. The *x*-axis shows the estimated insertion time (mya), estimated from the divergence level and mutation rate, while the *y*-axis shows the count of TEs inserted at each time interval

The repeat content of the newly assembled genomes is approximately 10–15% higher than previously reported for most snakes but consistent with the values reported for *H. curtus* (East) and *H. cyanocinctus* [6]. *Hydrophis major* exhibited the highest proportion of interspersed repeats (60.3% of the total genome length), followed by *H. elegans* (58%), *H. ornatus* (56.7%) and *H. curtus* (West) (56.2%) (Table 1; Additional file 2: Table S6), with all snakes sharing similar proportions of each repeat family (Fig. 2A–D). The repeat annotations were validated by running BUSCO on the hard masked genomes of each snake to ensure non-repetitive sequences were not misclassified. This saw complete BUSCOs only drop by approximately 1.3% relative to the unmasked genomes (Additional file 1: Fig. S11). Further, LTR Assembly Index (LAI) scores for all four assemblies exceeded the LAI gold-standard of LAI ≥ 20 [25] (Additional file 1: Figs. S12-15; Additional file 2: Table S7), indicating that not only are the repeat annotations accurate, but that these assessments likely indicate that transposable elements in *Hydrophis* snakes exceed 55% of the total genome sequence.

Repeat histories for the newly assembled *Hydrophis* snakes all show similar Kimura divergence profiles. Across all snakes, MITE elements account for a small percentage of the total genome and fall within the 15–25% divergence range, while LINE elements are slightly more abundant but appear to have been most active in the past (35–50% divergence), with some recent activity indicated by the bands observed between 15 and 20% (Fig. 2E). The largest repeat signals in all snakes come from DNA and long terminal repeat (LTR) elements. DNA elements show persistent activity in each of the snakes based on the breadth of their divergence profiles and consistent proportions. LTR elements are also abundant in each snake; however, historical activity that was observed in *H. major, H. curtus* (West) and *H. elegans* was not observed within *H. ornatus*, as indicated by the increase of LTR elements beginning at the 40% divergence range. Unique to *H. major* is a recent expansion of ‘Unknown’ repeats; these account for 3.4% of the total genome sequence (Fig. 2E) and consist of de novo modelled repeats along with repeat families from the curated DFAM and RepBase libraries.

In all the assembled sea snakes, LTRs were one of the most abundant repeat elements, accounting for 26.4% of all annotated interspersed repeats in *H. major*, 25.5% in *H. elegans*, 27.6% in *H. ornatus* and 27.3% in *H. curtus* (West) (Fig. 2A–D). Gypsy and Copia LTR elements constitute the predominant signal of LTR expansion in each of the four assemblies, with estimated insertion ages beginning ~ 12.5 million years ago (mya), with a peak in expansion occurring between 2.5 and 5 mya (Fig. 2F). The distribution and time of insertion is consistent with previous findings for *H. cyanocinctus* and *H. curtus* (see supplementary Table S4 in [6]). The insertion times and distribution of LTR elements in each of the four snakes are almost identical, which likely indicates shared expansions of these elements in the *Hydrophis* ancestor. However, in more recent time intervals, *H. major*,* H. curtus* (West) and *H. elegans* continue to share similar counts of Copia elements, while *H. ornatus* has had an expansion of these elements. In the same timeframe, *H. curtus* (West) had a dramatic increase in Gypsy elements that is not observed in any of the other snakes. Given the similarity across the rest of the distribution, it is likely that these represent recent species-specific expansions.

### Phylogenomic trees and networks

**Fig. 3:**
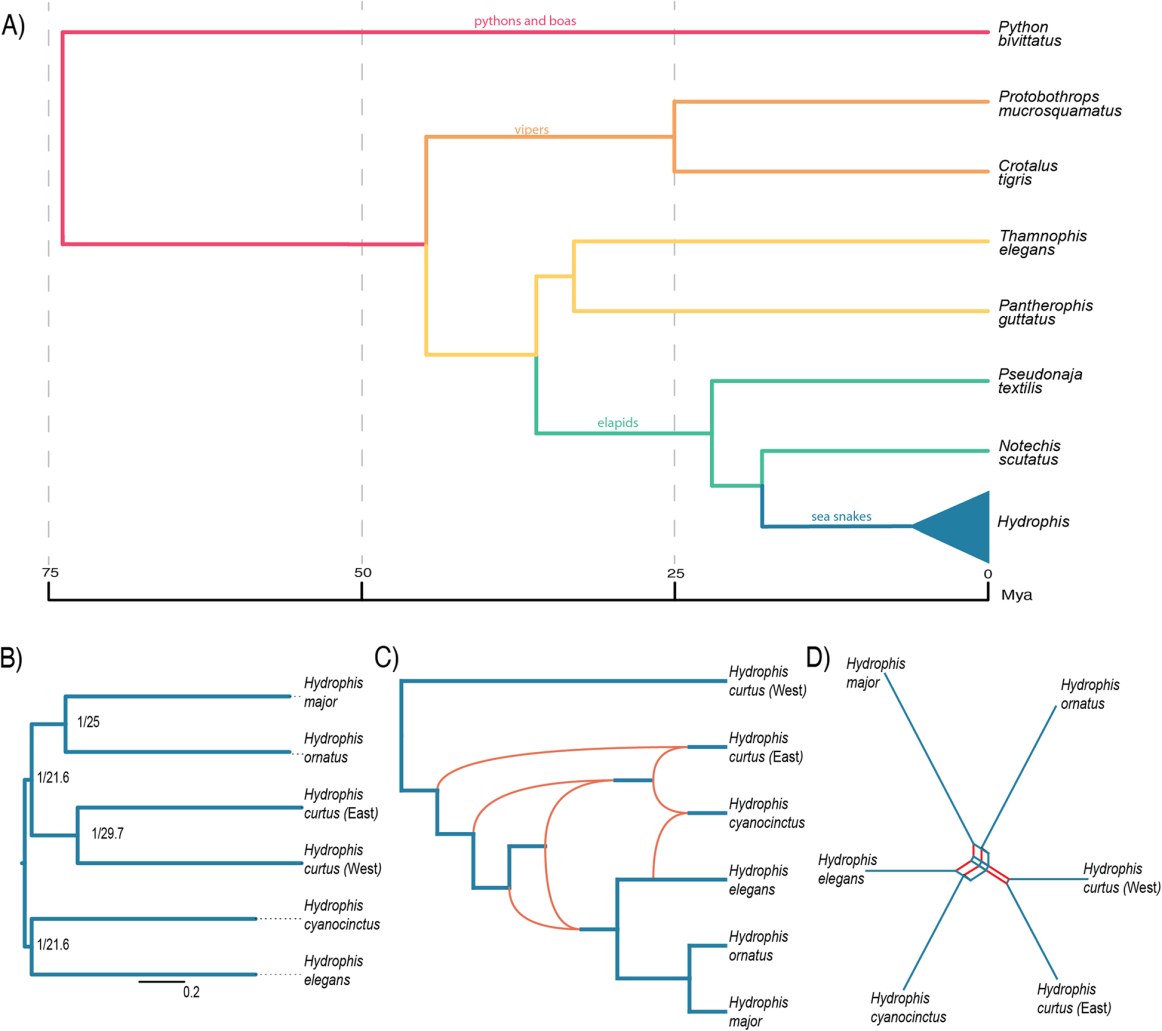
Species tree inference using *Hydrophis* single-copy orthologs and whole-genome sequences. **A** Time tree of sampled taxa drawn with Archaeopteryx 0.968 beta BG using relationships and divergence times from Lee et al. [11] and Zaher et al. [27]. **B** Species tree inferred from *Hydrophis*-specific single-copy orthologs using IQ-TREE and ASTRAL-III. Node labels are in the form ML support/gene concordance factor. **C** PhyloNet maximum likelihood network illustrating potential ILS/Introgression signals (orange arcs) between the six *Hydrophis* snakes. **D** A SANS serif weakly compatible splits network between the six *Hydrophis* snakes generated from the genome assemblies. Splits between snakes are represented by the red parallel edges

Our six *Hydrophis* species bridge the backbone of short, unresolved internal branches that characterise molecular phylogenies of the exceptionally rapid *Hydrophis* radiation [11, 26] (Fig. 3A). We therefore attempted to resolve their relationships using the newly generated genomes. A combination of phylogenomic tree and network analyses was performed to account for the possibility that the early radiation of *Hydrophis* species does not conform to a bifurcating tree.

Species tree and network analyses both resolved three reciprocally monophyletic lineage pairs: *H. curtus* (East) + *H. curtus* (West); *H. cyanocinctus* + *H. elegans; H. ornatus* + *H. major* (Fig. 3B, C)*.* Internal branches in the species tree are much shorter than the six terminal branches and are mirrored by edges of lower weight in the splits network (Fig. 3D). Species tree and network analyses yielded discordant internal relationships, with network analyses recovering *H. curtus* (East) + *H. curtus* (West) as sister to the other lineages pairs (Fig. 3C), while the IQ-TREE/ASTRAL-III species tree placed *H. cyanocinctus* + *H. elegans* as sister to the *H. curtus* lineages and *H. ornatus* + *H. major* with each node present in only ~ 20–30% of genealogies (Fig. 3B). Consistent with low gene tree concordance factors, network reconstruction using PhyloNet found support for a network over a tree (no reticulations) model, with model fit increasing with the number of reticulations specified (0 to 4) (Additional file 1: Fig. S16; Additional file 2: Table S8). The optimal network contained four reticulations (Fig. 3C) and was consistently recovered in replicate searches. Reticulations were concentrated early in the *Hydrophis* radiation and among the geographically overlapping *H. curtus* (East) and *H. cyanocinctus* lineages.

### Genome synteny

**Fig. 4:**
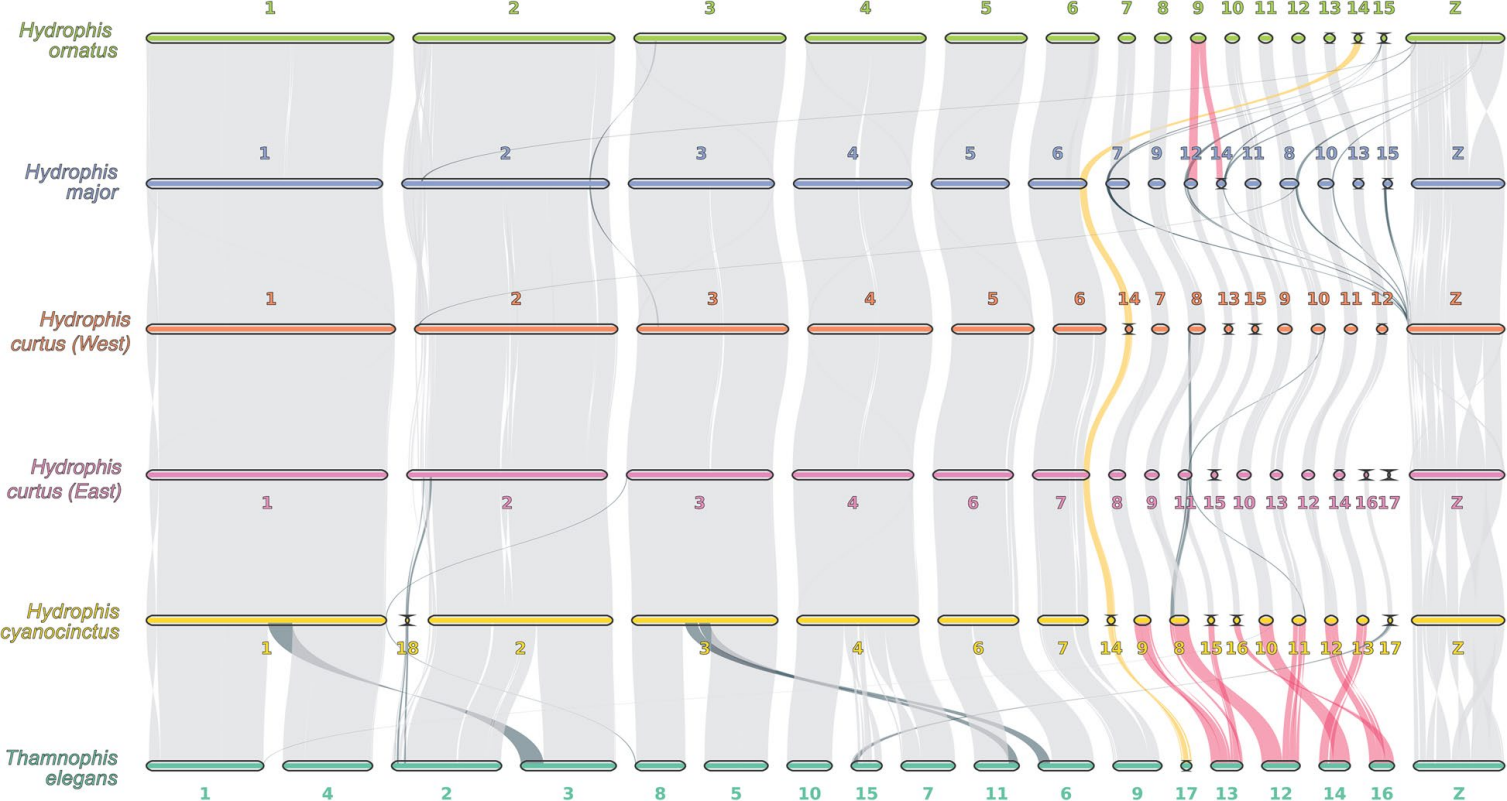
Synteny between the five chromosome-scale *Hydrophis* sea snakes and *Thamnophis elegans.* Chromosome sequences have been reverse transcribed in some instances to correct for strand variation between assemblies to improve interpretability (see Additional file 1: Fig. S16). Inter-chromosomal rearrangements are highlighted in dark-blue, chromosome fusion/fission events are in red and the chromosome 6 and 14 fusion/fission event which alternates between each of the *Hydrophis* snakes is in yellow

Genome-wide synteny analyses between the five chromosome-scale *Hydrophis* snakes, and outgroup *Thamnophis elegans*, were used to investigate karyotype, chromosome synteny, chromosome evolution and structural variation between these snakes. Chromosomal synteny between the *Hydrophis* snakes, both for macro- and micro-chromosomes, is extensive, with the karyotype varying slightly between snakes (Fig. 4; Additional file 1: Figs. S17 and S18). The substantial synteny between the *Hydrophis* snakes is not wholly unexpected based on their recent divergence (Fig. 3). Within the *Hydrophis* snakes, the six macro-chromosomes share broad homology, while the micro-chromosomes share significant homology but appear to be the main source of karyotypic variation. As reported in Li et al. [6], chromosome 14 exists as a micro-chromosome in *H. cyanocinctus,* as well as in *H. curtus* (West) and *H. ornatus* but is a part of a macro-chromosome 7 and 6 in *H. curtus* (East) and *H. major*, respectively. Chromosome 9 in *H. ornatus* appears to be a chromosome fusion of chromosomes 12 and 14 in *H. major*, or the two respective syntenic chromosomes in the four other *Hydrophis* snakes. Similarly, chromosome 8 in *H. cyanocinctus* looks to be formed from chromosomes 9 and 11 in *H. curtus* (East), with chromosome 11 in *H. curtus* (East) sharing homology to chromosome 15 in *H. cyanocinctus*. Some micro-chromosomes share limited-to-no homology to the other snakes, such as the *H. curtus* (East) chromosome 17, or its chromosome 16, which only shares homology with chromosome 17 in *H. cyanocinctus*. In *H. ornatus* and *H. major*, chromosome 15 shares homology to the Z-chromosome in all the other snakes, while chromosome 18 in *H. cyanocinctus* shares homology with the beginning of chromosome 2 in *H. curtus* (East), potentially reflecting a misassembly in *H. cyanocinctus*. Relative to the outgroup *T. elegans*, *Hydrophis* snakes share considerable homology, albeit across a vastly different karyotype. The five largest macro-chromosomes in *Hydrophis* all share homology to multiple different chromosomes in *T. elegans*, while the inverse is true for micro-chromosomes in *Hydrophis*, where multiple micro-chromosomes share homology to single chromosomes in *T. elegans* (Fig. 4). This suggests that there has been significant chromosomal evolution through time within elapid snakes, in the form of chromosome fission/fusion events, with *Hydrophis* snakes seemingly having settled on a relatively stable karyotype.

While broad syntenic patterns were observed between the *Hydrophis* genomes, we screened for structural variants (SVs) within *Hydrophis* using whole-genome alignments (Additional file 1: Fig. S18). Note that chromosome names used below refer to the chromosome identifiers used in Additional file 1: Fig. S18. Approximately 1.5Gbp of sequence was classified as syntenic between all pairs of species alignments, while unaligned regions accounted for anywhere between 130 and 300Mbp of the remaining sequence (Additional file 2: Table S9). Inverted regions were the most substantial SV in all species comparisons, affecting between 106 and 212Mbp of sequence in each genome comparison. The ends of chromosomes proved to be SV hotspots, with chromosome 1 housing a ~ 20mb inversion that alternates between all genome comparisons except *H. ornatus* and *H. major*, while the beginning of chromosome 2 has extensive SVs between all snakes. This region of chromosome 2 is dense with repetitive elements (Additional file 1: Fig. S18), perhaps indicating that its disorderly nature may be a result of repeat-activity, while also potentially reflecting the limitations of assembly methods in resolving such regions. Interestingly, between *H. curtus* (East) and *H. cyanocinctus*, small clusters of SVs were consistently observed towards the ends of all macro-chromosomes, a pattern not observed consistently in any of the other genome comparisons. Micro-chromosomes also proved to be littered with SVs, with chromosomes 11 and 12 harbouring significant proportions of SVs along their total length. However, the Z-chromosome is most notable, with nearly every portion of its sequence length being affected by a SV in at least one of the species comparisons.

### Gene selection during the marine transition of sea snakes

**Fig. 5:**
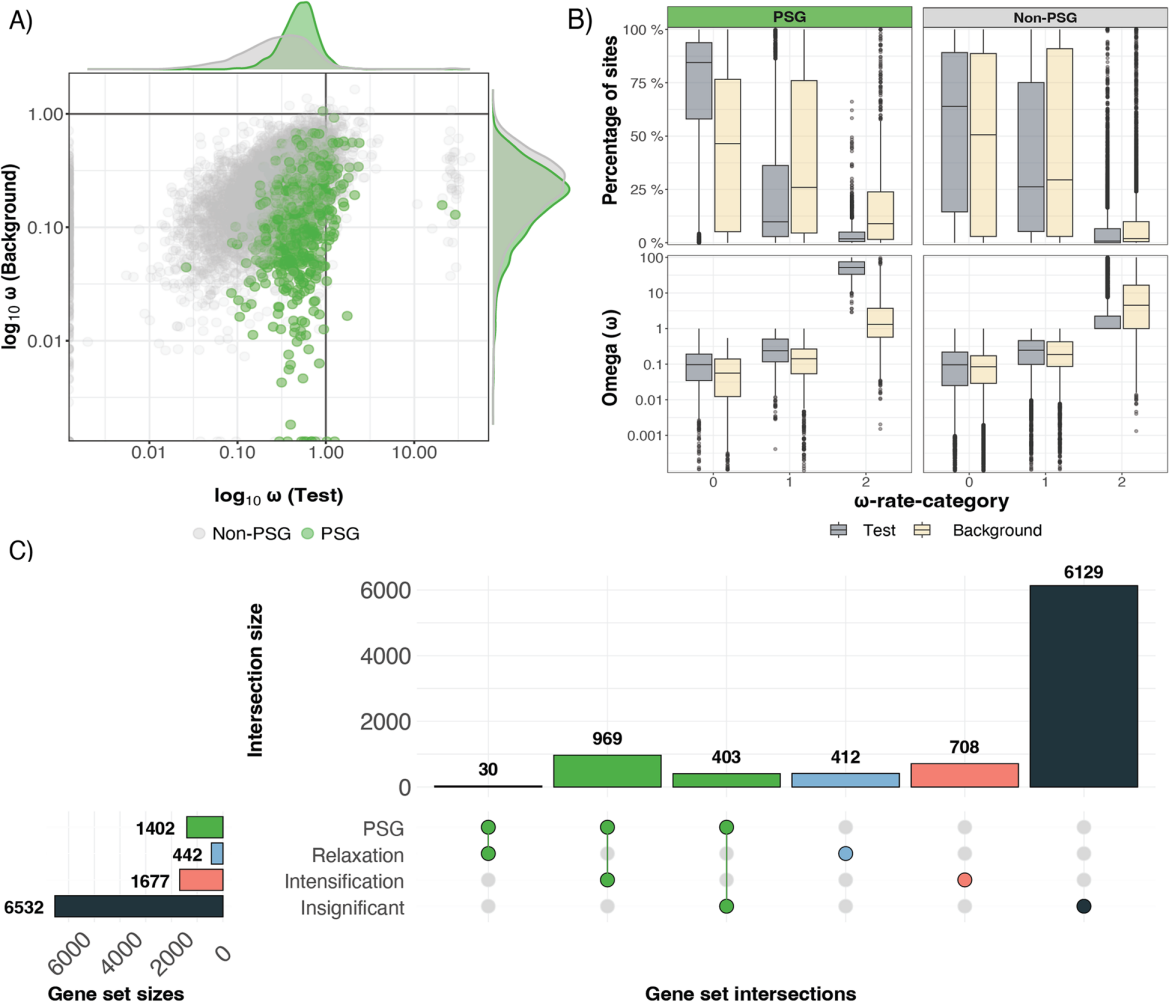
Exploration of selection testing results, along with the overlap between the marine positively selected genes (PSGs) and *RELAX* results. **A** Gene-wide *ω* values were computed during the BUSTED-PH analysis for each gene using the MG94xREV method. The *x*- and *y*-axes show the log_10_ transformed *ω* values for *Test* and *Background* branches, respectively. **B** Summary of the BUSTED-PH unconstrained model results (*ω* ≥ 1). The first facet column represents the marine PSGs (green) and the second facet column represents non-PSGs (grey). The top row shows the percentage of sites falling into each of the three *ω* rate classes, while the bottom row shows the distribution of *ω* values in each rate-class. **C** UpSet plot visualising intersections between the marine PSGs and each RELAX category (intensification, relaxation, insignificant). The central interaction matrix shows the combination of gene sets, with the top bar plot representing the size of the overlap. The left horizontal bar plot represents the size of the gene sets being compared

Using a set of 8654 single-copy orthologs obtained from thirteen snakes, we aimed to identify candidate genes associated with adaptive marine traits via their signal of positive selection within *Hydrophis* (Additional file 2: Tables S10 and S11). To identify marine-specific positively selected genes (PSGs), we used two selection testing methods that are both designed to identify signatures of positive selection that are trait specific and not phylogeny wide. Using the PAML drop-out method [28], we identified 2670 positively selected genes unique to *Hydrophis* after correcting for multiple testing (Additional file 2: Table S12), while the BUSTED-PH [29] approach reported 1608 genes as experiencing positive selection specific to *Hydrophis* (Additional file 2: Table S13). The final high-confidence gene set was obtained by intersecting the significant genes from each method, resulting in 1402 PSGs that were reported as only under positive selection within *Hydrophis* snakes (Additional file 2: Table S14). While there was considerable overlap in the genes identified by each method, the PAML approach identified an additional 1268 genes not reported by BUSTED-PH, whereas only 206 genes identified by BUSTED-PH were not also found by the PAML drop-out method (Additional file 1: Fig. S19). Mean *ω* ratios for single-copy orthologs were typically less than one, as is expected when averaging signatures of selection over branches and sites [30, 31], although mean *ω* values were notably higher for PSGs relative to the non-PSG set (Fig. 5A). This pattern can be explained by the proportion of sites in each rate-class and their respective *ω* values. Most sites in each ortholog belong to the purifying or nearly neutral *ω* rate categories (*ω* < *1* and *ω* ≤ *1*), with very few sites in the PSGs assigned to the third *ω* category (*ω* > *1*) (Fig. 5B; Additional file 1: Fig. S20). Consequently, the few positively selected sites with large *ω* values increase the overall average *ω* in the PSGs, even though the gene-wide average *ω* remains less than one. While there is evidence that some PSGs may also have experienced positive selection in the background lineages, indicated by the non-zero proportion of sites in the third *ω* rate category whose average *ω* is slightly above one (Fig. 5B), the overwhelming indication is that we have accurately identified a core set of genes that have only experienced positive selection in the *Hydrophis* lineages. Exploring the distribution of PSGs across the genome highlighted a relatively even spread, with PSGs residing on all chromosomes; however, no PSGs were identified in the tangle of SVs at the beginning of chromosome 2 (Additional file 1: Fig. S18).

In addition to identifying marine-specific PSGs, we also formally tested the strength of natural selection acting on the single-copy orthologs in *Hydrophis* relative to the terrestrial snakes using RELAX [32]*.* A total of 2119 single-copy orthologs were reported as significant after correcting for multiple testing (*FDR* ≤ *0.01*), of which 1677 reported an intensification of selection, 442 reported a relaxation of selection and 6532 remained insignificant (Fig. 5C; Additional file 2: Table S15). RELAX failed to run for three near-identical single-copy orthologs. Intersecting the RELAX results with the marine-specific PSGs showed that a large majority (969 of 1402) PSGs experienced an intensification of selection, 30 PSGs reported a relaxation of selection and 403 PSGs reported no significant intensification or relaxation of selection (Fig. 5C).

### GO term over-representation and semantic clustering

**Fig. 6:**
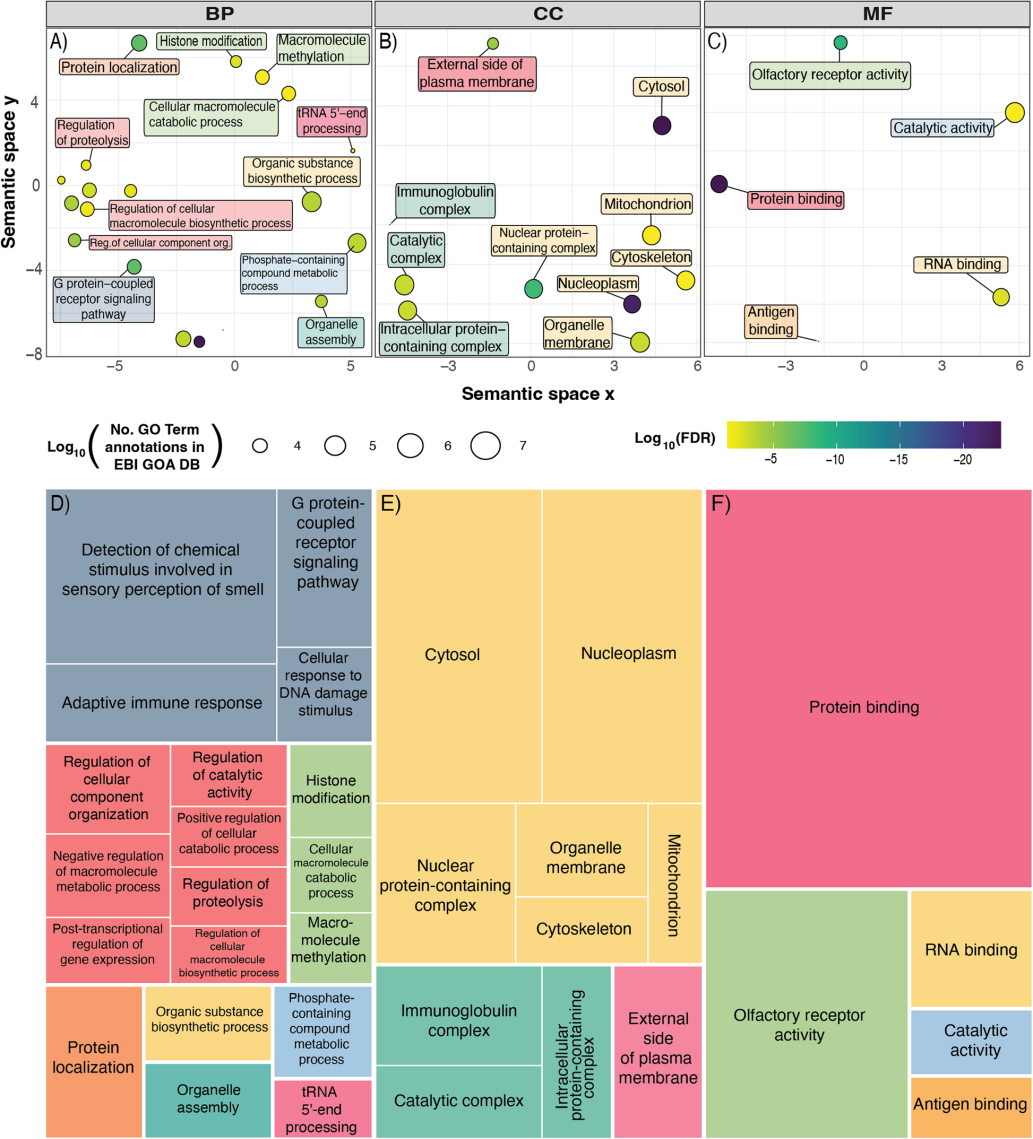
*REVIGO* multidimensional scaling (MDS) and TreeMap plots. **A-C** MDS plots for the Gene ontologies BP, CC and MF, respectively. The *x*- and *y*-axes represent arbitrary values for the semantic space. GO terms that are semantically similar cluster together. The colour of the circles represents the Log_10_(FDR) value, while the size of the circles represent the Log_10_ value of the number of annotations for the GO Term in the selected species in the EBI GOA database. Point labels have been coloured to match the TreeMap figures. **D-F** TreeMap figures generated by REVIGO*.* Semantically similar terms are clustered into broad categories, with the top-left term being the representative term for the group. Colours are ontology specific and do not match across ontologies

To understand the biological significance of the genes exhibiting positive selection in *Hydrophis*, we performed Gene Ontology (GO) under- and over-representation analysis using PANTHER [33, 34]. From the 1302 PSGs used to identify significantly over-represented GO terms (100 PSGs did not have annotated gene symbols), we identified 120, 26 and 12 GO terms as significantly enriched in biological process (BP), cellular component (CC) and molecular function (MF), respectively, after correcting for multiple testing (FDR ≤ 0.05) (Additional file 2: Table S16)*.* Taking the most specific enriched GO terms belonging to each hierarchical cluster resulted in a reduced set of terms belonging to each ontology (30 in BP, 10 in CC and 6 in MF). Of the over-represented terms, tRNA 5′-end processing (GO:0099116) had the highest fold enrichment, while other terms with increased fold enrichments related to catabolic processes within the cell (GO:0045732: positive regulation of protein catabolic process, GO:0031331: positive regulation of cellular catabolic process, GO:0044265: cellular macromolecule catabolic process, GO:0030162: regulation of proteolysis), regulation of transcription (GO:0010608: post-transcriptional regulation of gene expression, GO:0045893: positive regulation of DNA-templated transcription), metabolic processes relating to nitrogen and phosphate compounds (GO:0044271: cellular nitrogen compound biosynthetic process, GO:0051172: negative regulation of nitrogen compound metabolic process, GO:0006796: phosphate-containing compound metabolic process), circulatory system development (GO:0072359), animal organ development (GO:0048513) and cellular response to DNA damage (GO:0006974) among others (Additional file 1: Fig. S21; Additional file 2: Table S16). Under-represented terms were predominantly associated with sensory perception (GO:0004984: olfactory receptor activity, GO:0004930: G protein-coupled receptor activity, GO:0050911: detection of chemical stimulus involved in sensory perception of smell) and immunity (GO:0019730: antimicrobial humoral response, GO:0019814: immunoglobulin complex, GO:0003823: antigen binding; GO:0002250: adaptive immune response) (Additional file 1: Fig. S21; Additional file 2: Table S16). Semantic clustering of over- and under-represented GO terms using REVIGO [35] produced a reduced set of broad, related functional categories (Fig. 6A–F; Additional file 2: Table S17).

## Discussion

### Comparative and phylogenomics

Non-avian reptiles have emerged as an important group for studying karyotype evolution (reviewed in [36]). Our study contributes to this expanding literature by examining syntenic changes over a much more recent timeframe than most previous analyses of the group. The sequenced *Hydrophis* span less than 10 million years of evolution [37] and show extensive synteny across their macro- and micro-chromosome sequences. Most variation in karyotype is attributable to uncertainty in the assembly and identification of micro-chromosomes due to their unique sequence content, close proximity within the nucleus and propensity to interact with one another [38]. Chromosome counts within the assembled *Hydrophis* genomes are consistent with ancestrally reconstructed karyotype configurations [39] and earlier cytological work [40]. While snakes appear to have settled on a relatively stable ancestral chromosome configuration (2*n* = 36), the clade encompassing *Hydrophis* and another fully marine sea snake genus, *Aipysurus*, are both modelled as having a reduced karyotype (2*n* = 32). This deviation of karyotype in sea snakes is consistent with the reported increased rate of chromosome change within elapid snakes [41].

Structural variations, most commonly inversions, were observed along all *Hydrophis* chromosome sequences, with considerable variation along the total length of the Z-chromosome. Structural variants have been hypothesised to establish reproductive isolation and genomic diversity between incipient species by disrupting recombination, reducing introgression and potentially assisting in the development of genomic barriers during speciation [42, 43]*.* The intra-chromosomal variations identified here will be important targets for future adaptation and speciation genomic studies of this system.

The proportion of repetitive sequence content within squamate genomes varies greatly [44], with previous work on repetitive elements in snakes showing that their abundance in the genome is variable and that transposable elements likely contribute to increases in genome size [44–46]. Based on the six species of *Hydrophis* presented between this study and Li et al. [6], both genome size and repeat content appear relatively consistent at this phylogenetic scale. Galbraith et al. [46] showed that the proportion of repetitive elements in genome assemblies is strongly influenced by assembly quality, with short-read assemblies providing underestimations of genome size and repeat content due to collapsing recent, highly similar repetitive elements. This can be seen in *Hydrophis*; the initial draft genome of *H. curtus* had an assembly size of 1.62Gbp and repeat content of ~ 34% of genome length [7], before long-read technologies resulted in an assembly of 1.96Gbp with 55.6% of the genome being reported as repetitive [6]. As more high-quality genomes become available, it will be important to distinguish the effects of unequal assembly quality from genuine variation in genome size and repeat content across squamates.

Our phylogenomic analyses include only six of ~ 50 *Hydrophis* species, but these bridge the unresolved backbone of the genus and our results provide some new insights. Species tree and network analyses resolved two reciprocally monophyletic lineage pairs that have been recovered in previous analyses of mitochondrial and nuclear loci (two deep lineages within *H. curtus*, and a close affinity between *H. ornatus* and *H. major* with respect to the other sampled species), plus a sister relationship between morphologically similar species *H. cyanocinctus* and *H. elegans* that was not present in previous molecular trees [37]. The two approaches yielded discordant relationships with low concordance among genealogies. This topological conflict is despite maximal bootstrap and posterior support values for internal nodes, perhaps reflecting the tendency for support values to be inflated with increasing sampling of the genome [47]. The preferred network (versus tree) model recovered four reticulations that were mostly concentrated early in the *Hydrophis* radiation and among geographically overlapping lineages. It is likely that gene flow during speciation, post-speciation hybridisation and incomplete lineage sorting all contribute to these complex relationships, especially given the short intervals between speciation and large and overlapping populations within *Hydrophis* [48, 49]. However, it also remains to be determined whether the deepest speciation events in the clade conform to a bifurcating tree. *Hydrophis* presents a rich system for future studies to explore the challenges of distinguishing between weak phylogenetic resolution and near-simultaneous speciation.

### Gene selection during the land-sea transition of elapids

Our strict screens for gene selection in comparison to terrestrial snake genomes identified marine-associated genes in clusters of semantically similar terms related to marine phenotypes including hypoxia tolerance, sensory perception, immunity, osmoregulation and morphological traits. Below we explore our marine-associated genes in more detail, specifically in the context of marine adaptation in sea snakes and other secondarily marine vertebrates. This fills a conspicuous taxonomic gap in genomic studies of marine adaptation and has broad relevance for understanding how divergent and convergent changes to a shared genetic toolkit underpin ecological transitions in vertebrates.

#### Adaptations to hypoxia

The transition to a hypoxic and energetically demanding marine environment must have exerted significant pressures on the respiratory, cardiovascular and metabolic traits of sea snakes. In line with this expectation, GO terms such as *circulatory system development* (GO:0072359), *animal organ development* (GO:0048513) and *cellular response to DNA damage stimulus* (GO:0006974) were identified as being over-represented in the set of PSGs.

*Circulatory system development* (GO:0072359) contains genes related not just to cardiovascular and respiratory systems but also the circulation of nutrients and hormones. Several PSGs in this over-represented set, however, have specificities to cardiovascular and pulmonary function; for example *HAND2* and *SGCG* belong to the *dilated cardiomyopathy* KEGG pathway [50, 51] and epinephrine-binding receptor *ADRA2B* is a key gene in studies of broncho- and vasodilation in response to exercise in humans [52]. Selection on these genes might contribute to various aspects of the bimodal respiration of sea snakes. The respiratory tissues of the trachea and right lung extend almost the length of the sea snake body and the perfusion of these surfaces is enhanced by ‘anticipatory’ breathing tachycardia during surfacing [53]. Cutaneous gas exchange with seawater provides approximately a third of the oxygen requirements of sea snakes, and a significant portion of their CO_2_ and N_2_ loss [16, 17, 19, 54]. The skin is supplied by a dense network of capillaries, and a redistribution of blood flow (bypassing the lung) ensures the delivery of blood with favourable partial pressure gradients for gas exchange [9, 18]. Further facilitating gas exchange is modification of the permeability barrier of the skin [55, 56], specifically the inner layer composed of lipids and α-keratin [57, 58]. Our PSG set includes the α-keratin encoding *KRT24* gene, which has been lost independently in birds, crocodiles and mammals [59, 60] but may play a role in cutaneous respiration in snakes.

Respiratory adaptations of the blood are also prominent in studies of gene selection in secondarily marine taxa. Sea snakes, however, are reported to have haemoglobin concentrations, haemoglobin-oxygen affinities and haematocrit levels that are generally within the ranges reported for terrestrial snakes [19]. Nevertheless, PSGs linked to iron transport and homeostasis were included in several over-represented sets, including *nuclear protein-containing complex* (GO:0140513) and *organic cyclic compound binding* (GO:0097159). Heme-binding protein *HEBP1* and heme-exporter *FLVCR2* are involved in the regulation and transport of heme—an essential component of haemoglobin [61, 62]. Positive selection on these and other genes involved in heme biosynthesis and blood cell development suggests molecular adaptation involving oxygen storage and transport capabilities in sea snakes.

A notable sea snake PSG is lactate dehydrogenase A (*LDHA*). This is a key enzyme in the energy metabolism of hypoxia-tolerant species [63, 64]. It mediates pyruvate-lactate interconversion in blood, allowing the metabolism of lactate accumulated during periods of low oxygen availability. Selection on this gene in several fish and cetaceans is thought to increase energy production under hypoxic conditions by increasing affinity for pyruvate (which allows increased lactate production) [65, 66]. Relevant here is that *Hydrophis* also show positive selection on *SLC16A14*—a transporter of pyruvate and lactate across cells [67].

Six additional metabolism-associated PSGs (*NDUFA13*, *NDUFAF1*, *NDUFB1*, *NDUFV2*, *NDUFS7* and *NDUFAF3*) are nuclear-encoded components of the mitochondrial oxidative phosphorylation (OXPHOS) pathway responsible for generating ATP. These and other OXPHOS pathway genes have been reported to be under selection in several, independent lineages of marine-diving mammals (e.g. Tian et al. [66]).

Despite adaptations to meet oxygen demands, the fluctuating levels of hypoxia and reoxygenation experienced by diving animals causes a build-up of reactive oxygen species that damage DNA, proteins and lipids. There are 75 PSGs annotated to the enriched set *cellular response to DNA damage stimulus* (GO:0006974). Many of these genes are linked to hypoxia-induced oxidative stress and DNA damage repair, among other functions. Included in this PSG set are *DMAP1*, *SMARCAL1* and *USP7*, which are among twelve genes reported to be under positive selection for hypoxia tolerance in the high altitude adapted Tibetan hot-spring snake [68].

#### Salt and water balance

Sea snakes eliminate excess salts using specialised sublingual glands. Extrarenal salt glands have also evolved in birds, turtles and crocodiles [69] and use membrane transport proteins, notably the sodium–potassium pump (Na + /K + -ATPase), to transport ions across the epithelial cells lining the secretory tubules of the gland. Our PSG set includes *ATP1B4*, which encodes a subunit of the sodium/potassium-transporting ATPase [70]. However, this molecular pathway is involved in non-osmoregulatory functions, and altered selection on *ATP1B4* has been linked to traits such as toxin resistance in toads [71] and muscle development in tigers [72]. Expression studies are therefore needed to identify whether *ATP1B4* is upregulated in sea snake salt glands.

A PSG closely linked to osmoregulation is the renal chloride channel gene *CLCN5*, which encodes a member of voltage-gated chloride channel family that, in humans, is predominantly expressed in the kidney and plays a critical role in renal chloride reabsorption [73]. Selection on this and other renal genes indicates adaptation of kidney function in sea snakes despite observations of low concentrations and rates of excretion of salt in sea snake urine [74].

#### Neural and sensory adaptations

Related to sensory perception are under-represented terms *detection of chemical stimulus* (GO:0009593) and *G protein-coupled receptor activity* (GO:0004930), and one over-represented term—*anatomical structure development* (GO:0048856) (Fig. 6A, B; Additional file 1: Fig. S21; Additional file 2: Table S16). Many sensory PSGs relate to aspects of visual function. Among these are the aquaporin-encoding *MIP* gene, which has a specific and critical function in the transparency and refractive properties of the lens [75]. In aquatic vision, only the lens is responsible for focusing light onto the retina because the cornea has negligible refractive power in water [76]. Other vision PSGs include Retinitis Pigmentosa 2 (*RP2*), and Retinal Degeneration 3 (*RD3*), which function in the development of retinal photoreceptors [77–79]; *FSCN2*, involved in photoreceptor disk morphogenesis [80, 81]; *RDH10*, which encodes an enzyme that is involved in the production of retinoic acid in the retina [82]; and the eye development gene *TMX3*, which is also a candidate PSG in cichlid fish [83].

Sea snake PSG *BARHL1* is primarily expressed in the vertebrate cerebellum and is a key component of the proprioceptive pathway, which is the sense that provides information concerning movements, orientation and position of the body within the environment [84]. The proprioceptive capacity of sea snakes is virtually unknown but is likely to have been subject to selection during their transition to the three-dimensional marine environment. Three deafness-related PSGs might also perform roles in balance and spatial orientation: *PDZD7* and *GRXCR2* are involved in morphogenesis of the sensory hair cells (‘stereocilia’) in the inner ear [85, 86], and *TECTA* encodes a key protein of the tectorial membrane in which the stereocilia are embedded [87, 88]. These results provide the first candidate genes to complement morphological analyses of cerebellum [89] and inner ear [90] evolution during aquatic transitions in snakes.

Finally, several PSGs are associated with neural function and behaviour. These include *DCDC2*, which is linked to perception and memory [91, 92]; and *TIMELESS*, which regulates circadian rhythms [93, 94].

#### Morphological marine innovations

All sea snakes show major modifications of head, body and tail shape associated with marine locomotion and prey capture (e.g. Voris and Jayne [95]). However, *Hydrophis* show accelerated rates of head and body shape change that are linked to trophic specialisation, high sympatric diversity and elevated speciation rates compared to other sea snakes and terrestrial elapids [96]. These rapid morphological shifts in *Hydrophis* are underpinned by developmental mechanisms involving the presence and positioning of Hox boundaries and heterochronic changes in segmentation of the vertebrae [96, 97]. Our PSG set includes several genes that have key roles in cranial and axial patterning during embryonic development and are annotated to multiple over-represented GO terms. Three PSGs are bone morphogenetic proteins (*BMPER*, *BMPR1A* and *BMPR2*), which belong to a pathway of prolific candidate genes for morphological differentiation in numerous vertebrae systems (e.g. Darwin’s finches [98]; phyllostomid bats [99]). *BMPR1A*, for example, mediates craniofacial development, including tooth and palate formation [100–102]. Several PSGs belong to the Wnt signalling pathway (e.g. *AXIN2*, *PITX2*, *TCFL5*, *WNT4*, *WNT10A*, *WNT10B*, *WNT11*, *WNT2B*) and HOX family (*HOXA11 HOXD11*), both of which play critical roles in skeletal patterning [103–108]. Transcriptional studies will be needed to determine the specific roles of these candidate genes in the evolutionary development of sea snakes.

## Conclusions

We have generated four high-quality genomes for the prolific *Hydrophis* sea snake radiation. Three of these are at chromosome scale and represent the best reference genomes that have so far been provided for the medically important Elapidae based on assembly metrics relating to contiguity, base accuracy, sequence completeness and gene completeness. Our strict screens for gene selection in comparison to terrestrial snakes identified marine-associated genes in sea snakes that are linked to functional categories including hypoxia adaptation, sensory perception, immune response and morphological development. This fills a conspicuous taxonomic gap in genomic studies of marine adaptation and has broad relevance for understanding how divergent and convergent changes to a shared genetic toolkit underpin ecological transitions in vertebrates. Other results include phylogenomic tree and network analyses, which highlight the possibility of near-simultaneous speciation at the root of *Hydrophis*, and synteny maps showing intra-chromosomal variations that will be important targets for future adaptation and speciation genomic studies of this system.

## Methods

### Public genomes and sequence datasets

Existing genome assemblies and annotation files were downloaded for the following organisms: *Anolis carolinensis* [109], *Crotalus tigris* [110], *Naja naja* [111, 112], *Notechis scutatus *[113], *Pantherophis guttatus* [114], *Protobothrops mucrosquamatus *[115], *Pseudonaja textilis *[116], *Python bivittatus *[117] and *Thamnophis elegans* [118]. The genome assemblies of *Hydrophis curtus* (East) and *Hydrophis cyanocinctus* were also downloaded [6, 119, 120], along with their corresponding RNA-sequencing data [121, 122], which were filtered for contamination against the Kraken 2 (v2.1.2) standard database (dated 2021–05-17) [123] and quality filtered using Fastp (v0.23.2) [124].

### Sample collection

*H. major* and *H. elegans* were collected and sampled during fieldwork in Western Australia, using procedures approved by The University of Adelaide’s Animal Ethics Committee (approval number S-2015–119/34903), under a fauna taking licence (regulation 25, number FO25000393) granted by the Department of Biodiversity, Conservation and Attractions of Western Australia. *H. ornatus* and *H. curtus* (West) were sourced by collaborators from commercial fisheries operating in coastal waters of the Emirate of Fujairah, United Arab Emirates.

### Library construction and sequencing

PacBio HiFi sequence data was generated from high-molecular weight DNA from *Hydrophis major*. DNA was extracted from blood using the Monarch HMW DNA Extraction Kit for Tissue (New England BioLabs Inc.—#T3060S/L) as per the manufacturer’s instructions at Australian Genome Research Facility (AGRF) Adelaide. Two sequence libraries were constructed using the SMRTbell Express Template Prep Kit 2.0 (Pacific Biosciences, Menlo Park, CA, USA), which were each sequenced on a 8 M SMRT cell on a PacBio Sequel II system. HiFi circular consensus sequences (CCS) were generated from the subreads using the SMRT link software (v10.1.0.119588). Both the library construction and sequencing were performed at AGRF-University of Queensland PacBio facility (AGRF-UQ PacBio). The HiFi reads were then filtered for adapter contamination using the programme HifiAdapterFilt (v2.0.0) [125] (Additional file 2: Table S1). Hi-C libraries were prepared from kidney tissue using the Arima-HIC 2.0 protocol at the Australian Cancer Research Foundation Biomolecular Resource Facility (ACRF BRF). Libraries underwent size selection and were quality checked for concentration and size using Bioanalyzer and Qbit before being sequenced on an Illumina NovaSeq 6000 machine (2 × 150 bp paired end) (Additional file 2: Table S1). RNA from brain, skin, liver and vitellogenic follicles was extracted using RNeasy mini-kits (Qiagen), with sequence libraries prepared following the Illumina Stranded mRNA Prep, Ligation protocol (Illumina Inc., San Diego, USA). The libraries were then sequenced on an Illumina NovaSeq 6000 machine on S1 flowcells (NovaSeq 1.5 chemistry kits; 2 × 100 bp paired end). RNA-sequence data was then filtered for contaminants against Kraken 2 (v2.1.2) standard database (dated 2021–05-17) [123], with low-quality reads being filtered using Fastp (v0.23.2) [124] (Additional file 2: Table S1).

To generate Nanopore whole-genome data for *H. elegans*,* H. ornatus* and *H. curtus* (West), DNA was extracted from 25–50 μl of blood using the Circulomics Nanobind CBB Big DNA Kit, following the ‘Nanobind UHMW DNA extraction—Nucleated Blood Protocol’. Library preparation was performed on this DNA using the Ultra-Long DNA sequencing kit (SQK-ULK001) before the libraries were sequenced across 2 × promethION (FLO-PRO002) flow cells each for 72 h. DNase washes (EXP-WSH003) were performed at 24 and 48 h to help unblock pores and increase overall output. Basecalling was performed with Guppy (v4.0.11) for *H. ornatus* and *H. curtus* (West) and Guppy (v6.1.5) for *H. elegans* (Additional file 2: Table S1). For *H. ornatus* and *H. curtus* (West), 70 μl of blood was sent to ACRF Biomolecular Resource Facility, for generation of Hi-C libraries. Samples were prepared using the Arima Hi-C preparation and run on NovaSeq S1 300 cycles (2 × 150 bp Paired End), before trimming with Trim Galore (v0.6.6) [126] (Additional file 2: Table S1).

Short-read libraries were generated for *H. elegans, H. ornatus* and *H. curtus* (West). For *H. elegans*, DNA was extracted from tail tissue following the Gentra Puregene Tissue Kit protocol. DNA was sent to the South Australian Genomic Centre (SAGC), where libraries were generated according to Illumina DNA Prep (M) guidelines (Part No. 10000000254 v10) and Illumina to MGI Library Conversion (MGIEasy Universal Library Conversion Kit, Part No. MGI000004155), and were sequenced on a MGI DNBSEQ-G400 (2 × 150 bp paired end). The MGI sequence data was then filtered for contaminants and low quality against Kraken 2 and Fastp, respectively. For *H. ornatus* and *H. curtus* (West), DNA was extracted from blood using the Circulomics CBB Big Nanobind kit, following the ‘HMW DNA Extraction – Nucleated Blood’ protocol. DNA was sent to the Ramaciotti Centre for Genomics, where libraries were prepared using the Illumina TruSeq DNA PCR-Free workflow, and paired end sequencing was performed on a NovaSeq 6000 with a SP 2 × 150 bp flow cell. Reads were then trimmed using Trim Galore (v0.6.6) (Additional file 2: Table S1).

### Genome assembly

#### Genome size estimation

Genome size estimation was performed for each of the four sea snakes prior to assembly. High-accuracy sequence datasets for each snake (PacBio Hifi for *H. major*, Illumina for *H. ornatus* and *H. curtus* (West) and MGI for *H. elegans*) were passed to KMC (v3.2.1) to count 31-mers before being dumped to file using kmc_tools [127]. GenomeScope2 (v2.0) was then used to estimate genome sizes from the K-mer histograms [128].

#### Hydrophis major

The PacBio HiFi long reads were assembled into a primary and dual assembly (pseudo-haplotypes) using hifiasm (v0.16.1) [129, 130]. Hi-C reads were supplied to hifiasm to assist with phasing but were not incorporated into the initial assemblies. The Hi-C reads were then mapped to the primary and dual assemblies using an adapted version of the Arima Hi-C mapping pipeline. The cleaned Hi-C alignments were passed to pin_hic (v3.0.0) to perform iterative scaffolding and mis-join correction [131], followed by manual curation in Juicebox Assembly Tools (JBAT) to anchor scaffolds into chromosomes [132, 133]. Following manual curation, TGS-GapCloser (v1.0.3) was used to perform gap-filling [134], utilising the HiFi long reads, resulting in the final assemblies.

#### Hydrophis ornatus and Hydrophis curtus (West)

A total of 60 × Nanopore long reads and 70 × Illumina short reads and 50 × Hi-C reads were generated for both *H. ornatus* and *H. curtus* (West)*.* For assembly, initial contigs were generated from the ultra-long ONT reads using the Flye (v2.8.3) assembler [135], including 2 polishing iterations. The resulting contigs were then polished using Hypo (v1.0.3) [136], utilising both the Nanopore long reads and Illumina short reads. The programme Purge Haplotigs was then used to remove heterozygous, syntenic contigs from each of the primary assemblies to reduce redundancy before scaffolding [137]. The Hi-C data was processed with Juicer (v1.6) [133], then used as input for the 3d-DNA pipeline (v180419) [138]. The resulting assembly was then manually reviewed and edited in JBAT to form the final chromosome sequences for each snake.

#### Hydrophis elegans

Raw Nanopore reads were assembled into contigs using Flye (v2.9-b1768) [135]. Assembled contigs were then polished using the Nanopore data and the programme Medaka (v1.8.0) [139] before a final two rounds of polishing using the high-accuracy paired end sequence data via Nextpolish (v1.4.1) [140].

### Genome assessment

Multiple completeness measures were used to assess the quality of the four newly assembled genomes. General assembly metrics were generated using QUAST (v5.0.2) [141]. Reference-free assembly evaluation was performed with Merqury (v1.3) [142], comparing the k-mer profile of each assembly to its respective sequence dataset, generating k-mer spectra plots, k-mer recovery rate tables and Phred quality consensus estimates (QV) for each genome. Finally, the level of gene completeness in each assembly was assessed using BUSCO (v5.2.2) using the Tetrapoda (odb10) database of 5310 single-copy orthologs [143, 144].

### Repeat annotation

The de novo repeat annotation pipeline Extensive De Novo TE Annotator (EDTA; v2.0.1) was first used to model and annotate repetitive elements in each of the four snake genomes [145]. *EDTA* was run in ‘divide and conquer’ mode, first identifying LTR, TIR and helitron elements, before running the remaining annotation steps. To reduce the misclassification of gene sequences as repetitive elements, coding sequences from *Notechis scutatus* were provided as gene evidence from a somewhat evolutionarily close species. A combined library of EDTA modelled repeats and curated RepBase repeat sequences (v2018-10–26) were then passed to RepeatMasker to perform homology-based repeat annotation [146, 147]. Kimura divergence repeat landscapes were generated from the RepeatMasker output using the accessory script calcDivergenceFromAlign.pl and a custom R-script for visualisation. LTR insertion times (*T*) were estimated from the EDTA output using the equation *T* = *d/2μ*, where *d* is the sequence divergence between LTR pairs and μ is the mutation rate. The mutation rate 4.71 × 10^−9^ mutations per site/year was used for these calculations [6].

The quality of the annotated repeats were assessed using two approaches: by evaluating the loss of complete, single-copy BUSCOs from each genome after hard-masking repetitive elements, and via the LTR Assembly Index (LAI) [25], which is a formal measure of LTR-completeness within a genome assembly. The programme LAI, which is packaged with the programme LTR_retriever [148], was run on the output generated by EDTA.

### Gene annotation

The pipeline Funannotate (v1.8.11) was used to predict protein-coding genes in *H. major*, *H. cyanocinctus* and *H. curtus* (East) using transcriptomic, homology and de novo methods [149]. These snakes were de novo annotated as they all had transcriptomic data from the same sample that was assembled, and the Li et al. [6] snakes did not have public gene annotations at the time of writing. Prior to running the Funannotate pipeline, three external sources of supporting gene evidence were generated. First, Liftoff (v1.6.3) [150] was used to lift the RefSeq gene annotations from *Notechis scutatus*, *Psudonaja textilis*, *Naja naja*, *Protobothrops mucrosquamatus*, *Thamnophis elegans* and *Anolis carolinensis* to each of the three snakes. Next, protein sequences from the six RefSeq annotations above, the reviewed SwissProt database [151] and the lifted-over annotations from each snake were pooled and passed to MMseqs2 easy-cluster (v14.7e284) [152] to create a non-redundant set of representative proteins*.* This representative protein set, along with the reviewed Swissprot database, was then used to generate homology-based gene predictions for each snake using MetaEuk easy-predict (v6.a5d39d9) [153].

Funannotate train was then used to assemble genome-guided transcripts for each snake using Trinity (v2.8.5) [154], which were converted into transcript-derived gene models by PASA (v2.4.1) [155]. Gene prediction was then performed via the Funannotate predict module, which performs de novo and homology-based gene prediction, before incorporating the transcriptomic, de novo, homology and external sources of evidence into a non-redundant gene set using EVidenceModeler (v1.1.1) [156]. The external sources of gene and protein evidence were incorporated into the pipeline at this stage. Next, Funannotate update was used to refine the resulting gene models via two rounds of *PASA* annotation and compare, before filtering the gene set using expression information estimated by Kallisto (v0.46.1) [157]. The predicted gene models were screened against the InterProScan5 database (v5.57–90.0) [158] and EggNOG database (v5.0) using EggNOGG-mapper (v2.1.9) [159, 160] to obtain functional annotations, which were then compiled by Funannotate annotate into non-redundant functional annotations. As transcriptomic data was not available for the *H. ornatus*,* H. curtus* (West) and *H. elegans* samples, we elected to lift gene annotations from *H. major* to each of the three snakes using Liftoff [150]. Finally, BUSCO [143, 144] was used to assess the overall completeness of the predicted genes against Tetrapoda (odb10), while length distribution plots were used to compare the predicted gene models to the RefSeq annotated snakes.

### Species tree estimation

A combination of phylogenomic tree and network analyses was performed to account for the possibility that the early rapid radiation within *Hydrophis* does not conform to a bifurcating tree.

First, a two-step approach was used to build a species tree while accounting for discordances with and among gene trees. Maximum likelihood gene trees were inferred for 9277 *Hydrophis*-specific single-copy orthologs, all of which had at least one parsimony-informative site, using IQ-TREE (v2.2.0.3) [161]*.* For each ortholog, IQ-TREE was allowed to automatically select the best-fitting model and performed 1000 ultrafast bootstraps. These individual trees were then used to infer a single species tree with ASTRAL-III (v5.7.8) [162]. To explore discordance among gene trees, we calculated gene concordance factors (gCF) using IQ-TREE, which represent the proportion of gene trees that contain each node of the species tree.

Species networks were inferred from a reduced set of IQ-TREE gene trees using PhyloNet (v3.8.2) [163] with the command InferNetworks_ML. Network searches were run with 5 iterations, allowing up to 4 reticulation events, with branch lengths and inheritance probabilities optimised for each proposed network. To reduce run times, and due to the limited amount of sequence variation per ortholog, the IQ-TREE gene trees were filtered using PhyKIT (v1.11.15) [164] to find the set of 2568 trees that included all six taxa and at least five parsimony-informative sites.

Finally, the full *Hydrophis* assemblies were used as input to SANS serif (v2.3_3A) [165], which calculates a set of splits by comparing shared k-mer distributions between unaligned reference genomes. SANS serif was run using the default k-mer value (*k* = 31), the geometric mean weight function and using the *weakly* filtering criterion to greedily filter the list of splits.

### Genome synteny and structural rearrangements

Broad-scale genomic synteny was investigated between the five chromosome-scale *Hydrophis* assemblies (*H. ornatus, H. major, H. curtus* (West)*, H. curtus* (East) and *H. cyanocinctus)* and the chromosome-scale outgroup *Thamnophis elegans* using MCscan [166]. Protein sequences from each snake were extracted using AGAT (v0.9.2) [167] and were pairwise aligned using LAST [168] using the JCVI python module [169]. Initial alignments between snakes were used to identify chromosomes assembled in the reverse complement, which were corrected in one of the snakes by reverse complementing the sequence using SAMtools faidx (v1.16.1) [170]. Gene annotations were lifted over to the newly reversed sequences using Liftoff, before re-running the MCscan pipeline to generate the final, oriented synteny plots.

Structural variation between the *Hydrophis* snakes was assessed using the programme Syri (v1.6.3) [171]. The curated genomes used in the MCscan alignments were further edited to have the same karyotype and chromosome identifiers (a requirement of Syri). *Hydrophis ornatus* was used as the anchoring reference, with the karyotypes and chromosome identifiers of the other *Hydrophis* snakes edited to match. Pairwise alignments were then performed between the snakes using Minimap2 (v2.24-r1122) [172], with the resulting alignment files passed to Syri to identify structural variation. Plotsr (v1.1.0) was then used to visualise the structural rearrangements in a single plot [173].

### Single-copy ortholog detection

The snakes *Hydrophis major, H. elegans, H. ornatus, H. cyanocinctus, H. curtus* (East)*, H. curtus* (West)*, Crotalus tigris, Notechis scutatus, Pantherophis guttatus, Protobothrops mucrosquamatus, Pseudonaja textilis, Python bivittatus *and* Thamnophis elegans* were used for single-copy ortholog identification. Snakes not part of this study were selected due to having RefSeq gene annotations and a minimum *BUSCO* score of > 85%. For each snake, AGAT (v0.9.2) was used to extract each gene’s longest isoform in peptide and nucleotide format. OrthoFinder (v2.5.2) was then used to find orthogroups, using the protein sequences and estimated species tree (see above) [174]. Single-copy orthologs were then aligned using Mafft (v7.505) [175] and codon-translated using Pal2Nal (v14) [176], before dynamic trimming using ClipKIT (v1.3.0) [177]. Orthogroup annotations were generated from a range of data sources. Gene symbols were parsed from RefSeq and Funannotate gene annotation files, along with BLASTP (v2.12.0) [178] results after screening protein sequences against SwissProt [179].

### Selection testing

#### Signatures of positive selection in Hydrophis

Two separate techniques were used to identify *Hydrophis*-specific signals of positive selection using the single-copy orthologs: PAML drop-out experiments and BUSTED-PH.

PAML is a maximum likelihood method that infers positive selection using the non-synonymous/synonymous rate ratio (*dN/dS* or *ω)*, where *ω* > 1 is indicative of positive selection, *ω* < 1 signifies purifying selection and *ω* ≈ 1 is neutral [30, 180]. PAML Branch-Site tests for positive selection were run for each single-copy ortholog, with the *Hydrophis* snakes (and all subtending branches) marked as foreground. To account for signatures of pervasive selection (i.e. tree-wide selection), we performed drop-out tests as recommended by Kowalczyk et al. [28], whereby foreground branches were removed from the species tree and Site models were run for each ortholog on the background branches. Likelihood ratio tests (LRT) were then used to compare the null and alternate models within the Branch-Site and Site tests, respectively, with a Benjamini–Hochberg correction being applied to account for multiple testing (FDR ≤ 0.01) [181]. Genes were only considered as under positive selection within *Hydrophis* when the Site model failed to reach significance (FDR > 0.01), but the Branch-Site did (FDR < 0.01).

The second approach involved running the programme BUSTED-PH for each single-copy ortholog. BUSTED (Branch-site Unrestricted Statistical Test for Episodic Diversification) is a model which provides a gene-wide test for positive selection, asking if a gene has experienced positive selection at least one site along at least one branch [31]. The workflow BUSTED-PH (BUSTED-Phenotype) builds on this model by testing if a specific phenotype/trait is associated with positive selection by performing a series of selection tests that are similar to the drop-out tests above [29]. BUSTED-PH not only uses a different selection testing framework, but it also provides context relating to the selective regimes between the *Test* (*Hydrophis*) and *Background* (*Terrestrial*) species. BUSTED-PH was run for each single-copy ortholog using the same tree partitioning scheme as the PAML drop-out analyses, with the same FDR correction being applied (FDR ≤ 0.01). The final set of *Hydrophis*-specific positively selected genes was obtained by taking only genes that reached significance in both methods (FDR ≤ 0.01) and showed no sign of pervasive selection across the tree.

#### Testing the strength of natural selection

Understanding the selective regime of genes is equally as important as simply identifying selection candidates. RELAX is a hypothesis testing framework for detecting selective strength in a codon-based phylogenetic framework [32]. It is a relative measure, testing if the strength of selection in a *Test* partition is different to that of the *Reference* group. It first fits a null model where the *Test* and *Reference* branches share the same selective regime, before fitting the alternate model which incorporates a selection intensity parameter—*k*—a free parameter that is used to adjust the *Test* ω rate classes while being fixed to *k* = 1 in the *Reference* set. The null and alternate models are then compared using an LRT statistic, with a significant LRT statistic indicating differing selective regimes between *Test* and *Reference* branches. The strength of selection is then interpreted from the selection intensity parameter* k*, where *k* > *1* represents an intensification of positive selection and *k* < *1* indicates a relaxation of positive selection. RELAX was run on each single-copy ortholog using a species tree where *Hydrophis* snakes were marked as the *Test* and all remaining branches marked as *Reference*. FDR corrections were applied to LRT *p*-values using a filtering criterion of FDR ≤ 0.01.

### Gene ontology over-representation and semantic similarity analysis

Gene ontology over-representation analysis was used to identify biologically relevant GO terms within the *Hydrophis* positively selected gene set. The programme PANTHER (*GO database released 10 May 2023*) was used to perform the over-representation tests [33, 34], to identify functional classes that were significantly over- or under-represented in the input list of genes. The *Hydrophis* PSGs were passed to PANTHER to perform the over-representation analysis, specifying *Homo sapiens* as the reference organism, using Fisher’s exact test with FDR multiple test correction (*FDR* < 0.05) to perform the over-representation test. Significantly enriched GO terms were returned as a hierarchical table, with the most specific GO Terms being reported first, followed by more general parental terms occurring as nested entries. The programme REVIGO (GO database released 10 May 2023; UniPort-to-GO mapping database released 15 March 2023) was then used to reduce the over-represented GO terms into an informative set of non-redundant, representative biological terms [35]. Over-represented GO terms and their *p*-values were passed to REVIGO, setting the semantic threshold to ‘small’, filtering obsolete GO terms and setting UniProt as the database to compare to.

### Supplementary Information

**Additional file 1:** **Fig. S1.** Cumulative length of assembled sequences. **Fig. S2.** Assembly Nx plots. **Fig. S3.** *H. major* Hi-C contact map. **Fig. S4.** *H. curtus *(West) and *H. *cyanocinctus Hi-C contact maps. **Fig. S5.** Genome size estimation. **Fig. S6.** *H. major *k-mer spectra and multiplicity. **Fig. S7.** K-mer spectra for *H. elegans, H. curtus *(West) and *H. cyanocinctus.* **Fig. S8.** Assembly BUSCO completeness. **Fig. S9.** Length distributions of gene features. **Fig. S10.** Protein BUSCO completeness.** Fig. S11.** Hard-mask BUSCO completeness. **Fig. S12.** *H. *major sliding window LAI scores. **Fig. S13.** *H. ornatus* sliding window LAI scores. **Fig. S14.** *H. curtus* (West) sliding window LAI scores. **Fig. S15.** *H. elegans* sliding window LAI scores. **Fig. S16.** PhyloNet networks for varying reticulation values.** Fig. S17.** Chromosomal synteny before manual curation. **Fig. S18.** Structural variation between *Hydrophis* snakes. **Fig. S19.** Overlap between PAML drop-out and BUSTED-PH methods. **Fig. S20.** Summary of PAML branch-site (alternate model) results. **Fig. S21.** Fold enrichment reported by PANTHER.

**Additional file 2: Table S1.** Sequence data summary. **Table S2.** Estimated genome size statistics. **Table S3.** Genome assembly summary metrics. **Table S4.** K-mer completeness and QV statistics.** Table S5.** Gene annotation summary statistics. **Table S6.** RepeatMasker summary tables. **Table S7.** LTR Assembly Index results for each of the newly assembled genomes. **Table S8.** PhyloNet output for each reticulation value. **Table S9.** A summary of structural variants identified by Syri between sea snakes. **Table S10.** OrthoFinder summary statistics. **Table S11.** Orthogroup summary statistics. **Table S12.** PAML drop-out results for each single-copy ortholog. **Table S13.** BUSTED-PH results for each single-copy ortholog. **Table S14.** Selection testing results for marine-specific positively selected genes. **Table S15.** RELAX results for each single-copy ortholog. **Table S16.** Over-representation results generated by PANTHER. **Table S17.** REVIGO clustering of the 46 most specifically enriched GO terms.

## Data Availability

All data generated or analysed during this study are included in this published article, its supplementary information files, and publicly accessible repositories. Genome assemblies for *H. major* (JAUCBL000000000) and *H. elegans* (JAUPSR000000000) have been uploaded to NCBI GenBank under the BioProject accession PRJNA984433 [182]. Raw sequencing reads generated for *H. elegans* have additionally been uploaded to the NCBI Sequence Read Archive (SRA) under the same BioProject. Raw sequencing data for *H. major* is available through the Bioplatforms Data Portal under the following dataset identifiers: PacBio HiFi (102.100.100/351827 and 102.100.100/351778), Hi-C (102.100.100/351780) and RNA (102.100.100/351777). Genome assemblies and sequencing data (Nanopore, Illumina and Hi-C) for *H. ornatus* (JAWKAR000000000) and *H. curtus* (West) (JAWKAS000000000) have been uploaded to NCBI GenBank and SRA under BioProject PRJNA780942 [183]. Scripts and pipelines used to conduct the analyses are available through GitHub [184], with additional data files deposited to figshare [185].

## Abbreviations

QV: Consensus quality value
LAI: LTR Assembly Index
LTR: Long terminal repeat
MYA: Million years ago
SV: Structural variant
PSG: Positively selected gene
GO: Gene Ontology
BP: Biological process
CC: Cellular component
MF: Molecular function
MDS: Multidimensional scaling
SRA: Sequence read archive
