# Supplementary material for: New chromosome-scale genomes provide insights into marine adaptations of sea snakes (Hydrophis: Elapidae)
Source: BMC Biol. 2023 Dec 8;21:284. doi: 10.1186/s12915-023-01772-2 (PMC10709897; doi:10.1186/s12915-023-01772-2)
Supplement: Supplementary file 1 — Additional file 1: Fig. S1. Cumulative length of assembled sequences. Fig. S2. Assembly Nx plots. Fig. S3. H. major Hi-C contact map. Fig. S4. H. curtus (West) and H. cyanocinctus Hi-C contact maps. Fig. S5. Genome size estimation. Fig. S6. H. major k-mer spectra and multiplicity. Fig. S7. K-mer spectra for H. elegans, H. curtus (West) and H. cyanocinctus. Fig. S8. Assembly BUSCO completeness. Fig. S9. Length distributions of gene features. Fig. S10. Protein BUSCO completeness. Fig. S11. Hard-mask BUSCO completeness. Fig. S12. H. major sliding window LAI scores. Fig. S13. H. ornatus sliding window LAI scores. Fig. S14. H. curtus (West) sliding window LAI scores. Fig. S15. H. elegans sliding window LAI scores. Fig. S16. PhyloNet networks for varying reticulation values. Fig. S17. Chromosomal synteny before manual curation. Fig. S18. Structural variation between Hydrophis snakes. Fig. S19. Overlap between PAML drop-out and BUSTED-PH methods. Fig. S20. Summary of PAML branch-site (alternate model) results. Fig. S21. Fold enrichment reported by PANTHER. [file 12915_2023_1772_MOESM1_ESM.docx]

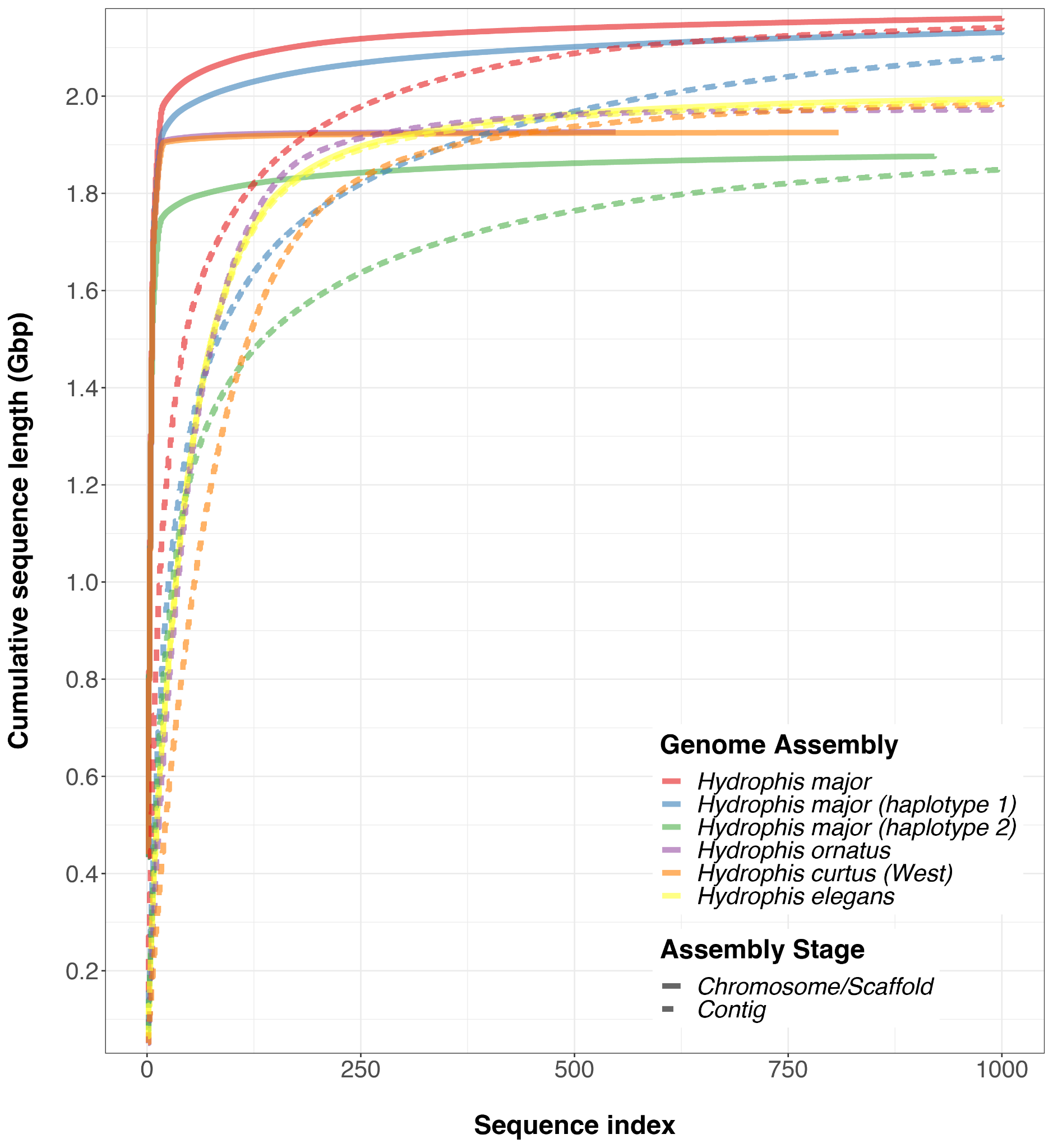


**Fig. S1**: Cumulative length of all assembled sequences (chromosomes, scaffolds and unplaced scaffolds). Assembled sequences are indexed from longest-to-shortest (x-axis), with the cumulative total length of the assembled sequences plotted along the y-axis. The final assemblies (chromosome/scaffold level) are represented by solid lines, with the initial contig assemblies represented by dashed lines.


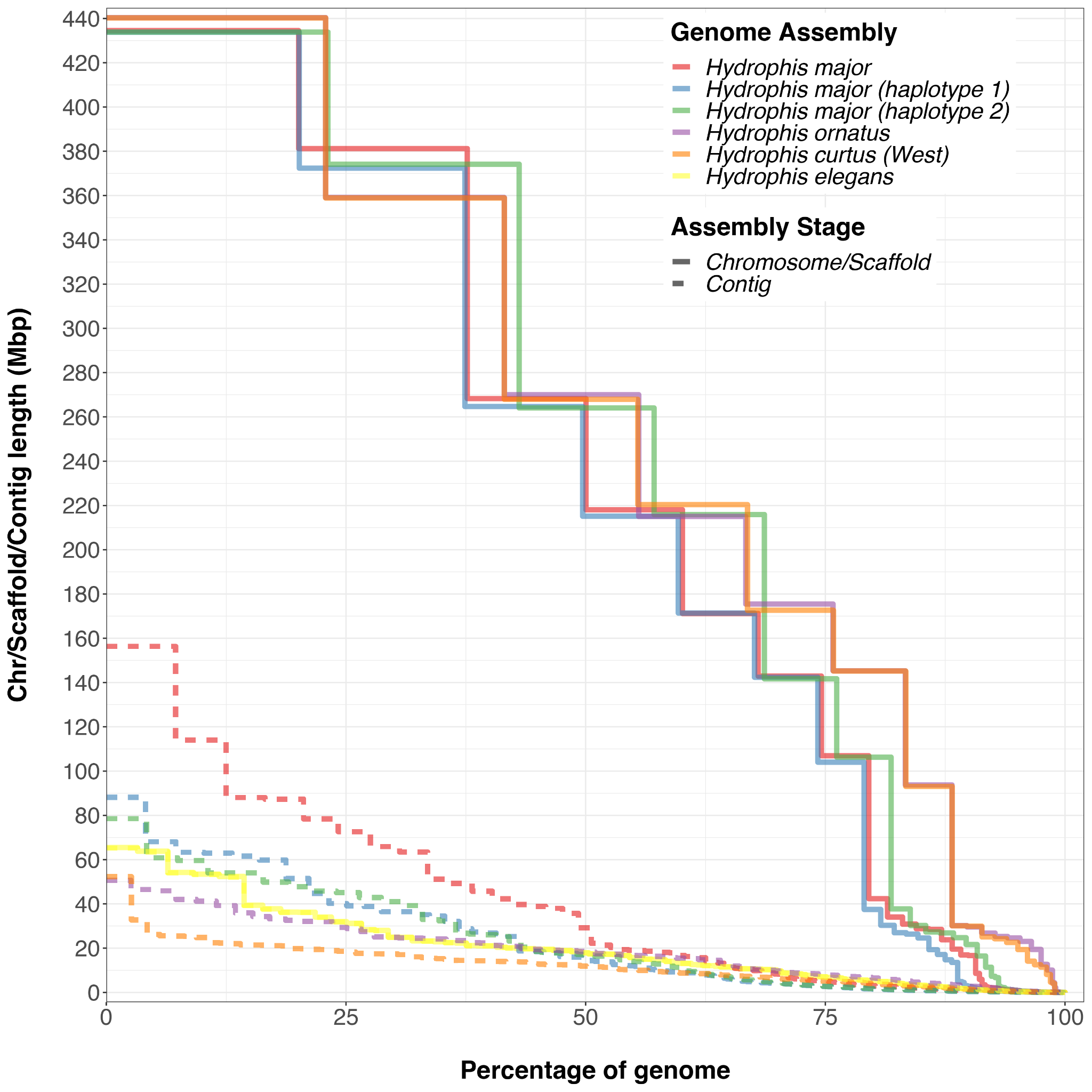


**Fig. S2:** Nx plot of assembled genomes representing overall contiguity. The figure displays the percentage of the genome (x-axis) that is composed of sequences of a minimum length or longer (y-axis). Initial contig assemblies are represented by the dotted lines, with the solid lines representing the final scaffold/chromosome scale assemblies.


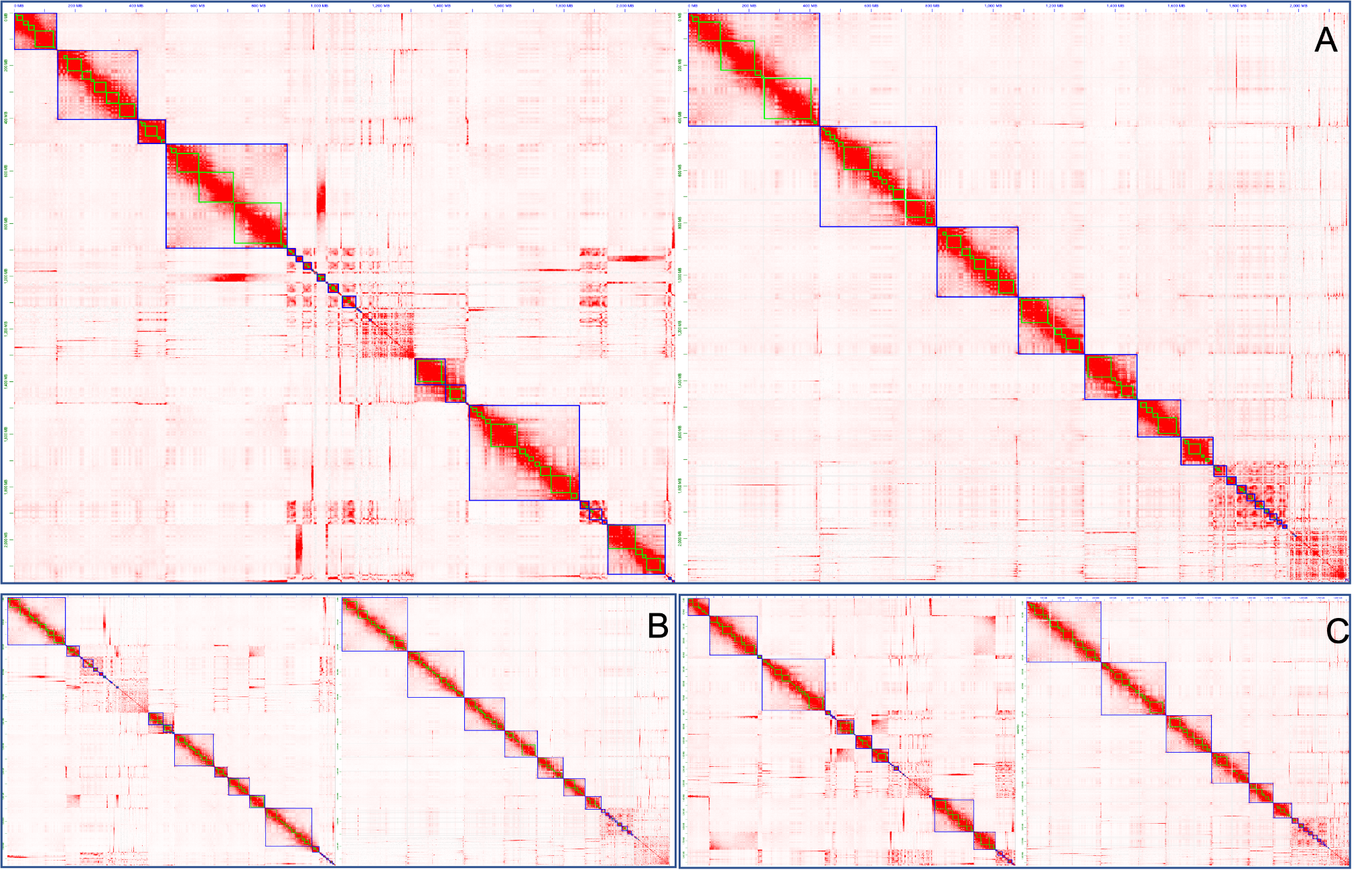


**Fig.S3:** Hi-C contact maps before and after manual curation for the Hydrophis major primary and haplotype assemblies. A) The pin_hic contact map for the primary contig assembly of *H. major*. The contact map on the left represents the raw contact before manual orientation via JBAT. The figure on the right represents the manually ordered contigs forming the chromosome scaffolds. Figures B) and C) represent the same process for haplotype-1 and haplotype-2 of *H. major*, respectively.


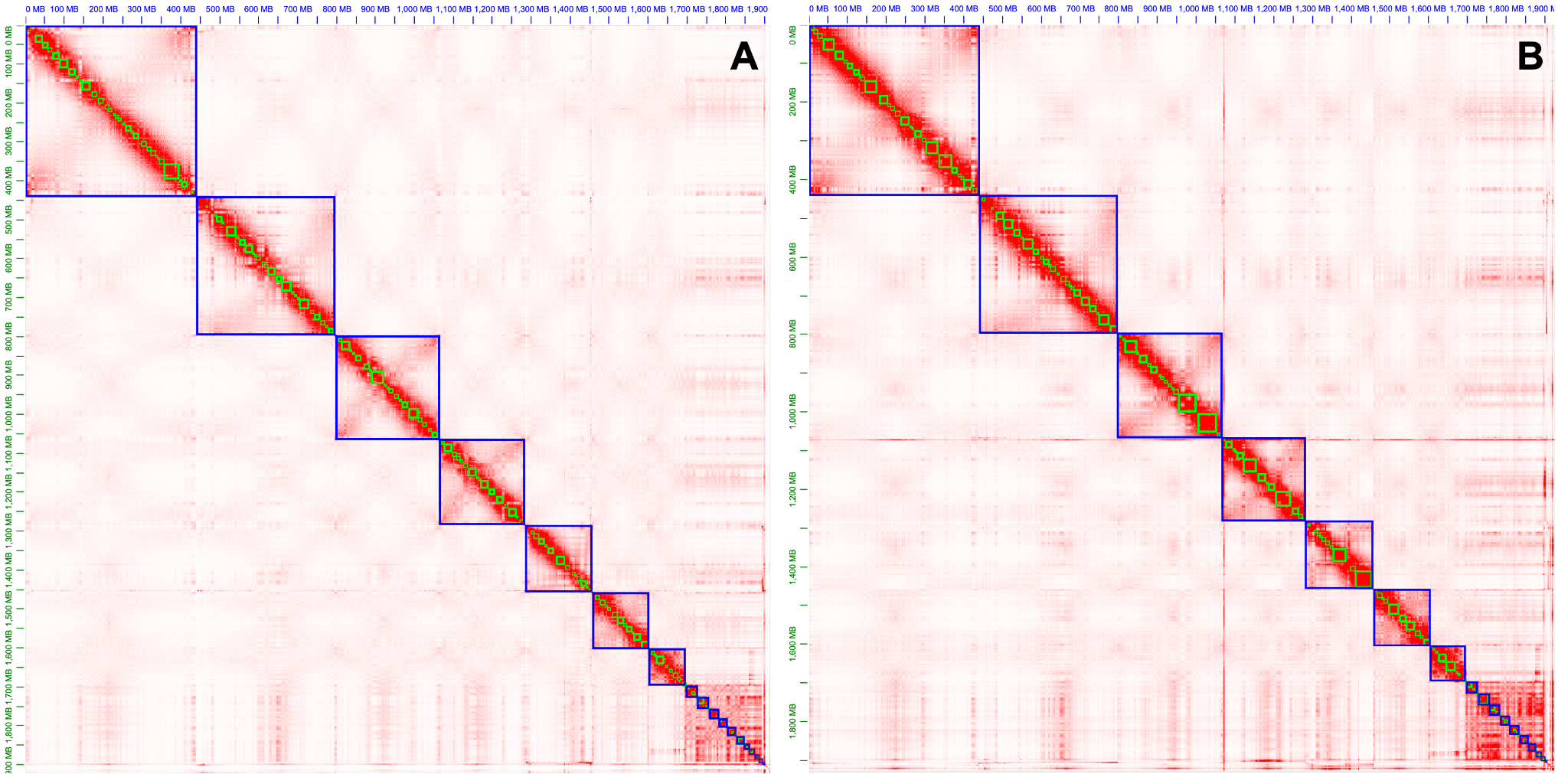


**Fig. S4:** Hi-C contact maps for (A) *Hydrophis curtus (West)* and (B) *Hydrophis ornatus*. Assembly files from 3d-DNA were edited in *JBAT* to form the final chromosome sequences for each snake.


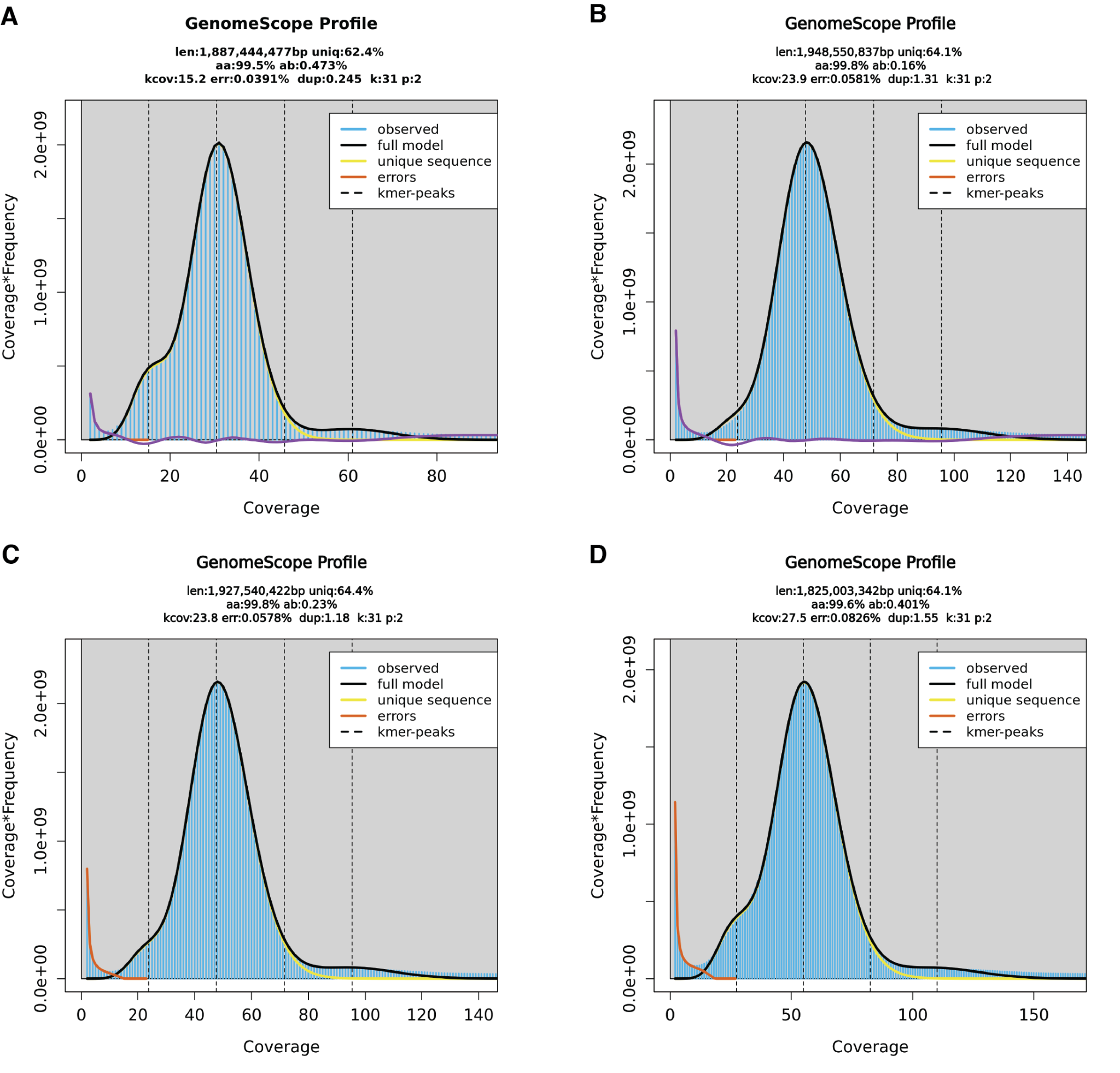


**Fig. S5:** Genome size estimation. *GenomeScope2* profiles for the four snakes assembled in this study. The main peak in each figure represents the homozygous portion of each genome, with the center of the peak corresponding to the approximate coverage of the homozygous portion of the genome. The shoulder to the left of the main peak corresponds to the heterozygous portion of the genome. A) Genome profile for *Hydrophis major* B) *H. ornatus* C) *H. curtus* (West) and D) *H. elegans.*


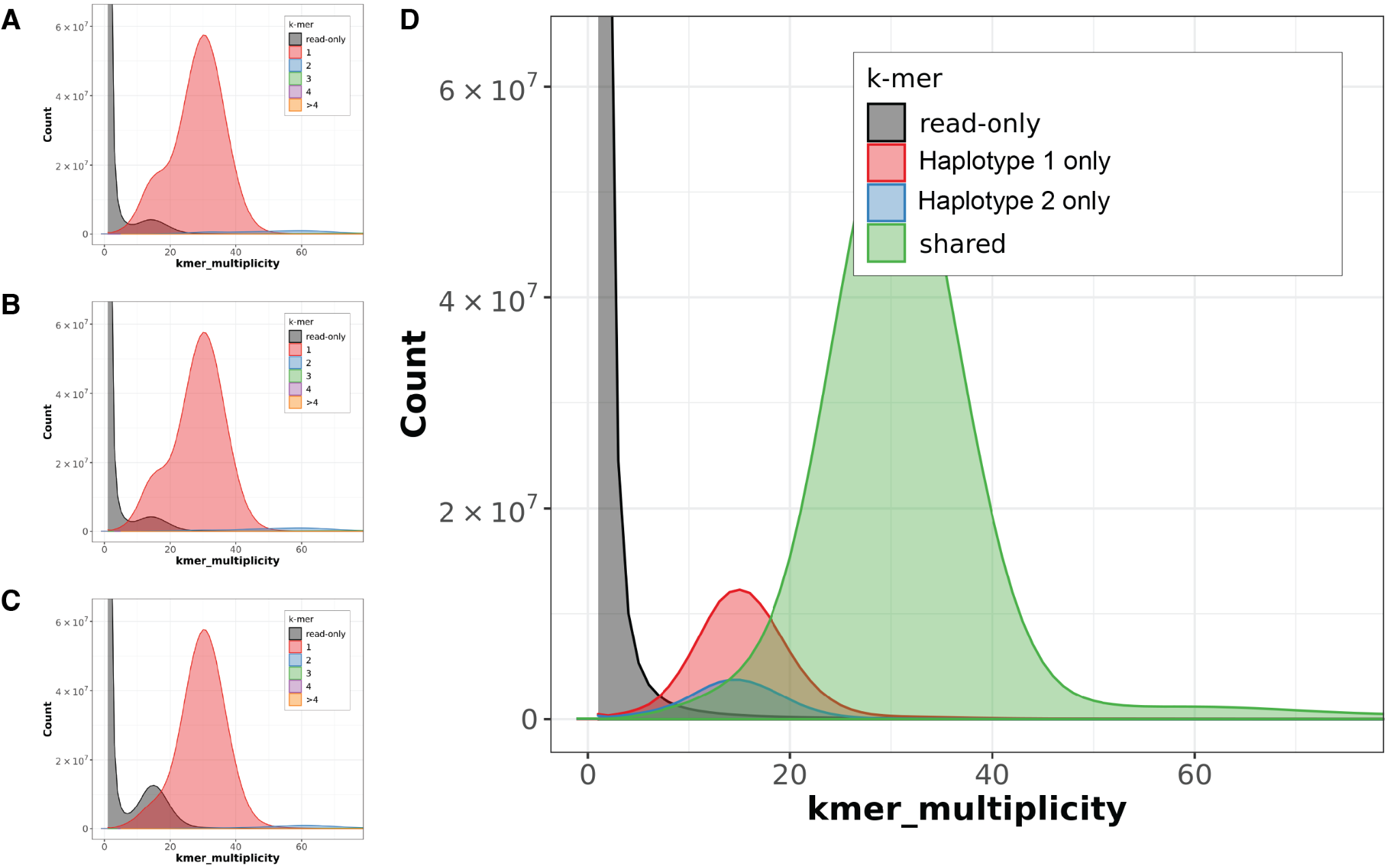


**Fig. S6:** Merqury k-mer spectra analysis for *H. major*. A-C) Per-assembly spectra plots showing the k-mer multiplicity in the primary assembly, haplotype-1 and haplotype-2, respectively. K-mers appearing in the genome once are in red, twice are in blue, three times in green and four or more times in purple and orange respectively. D) Assembly spectra plot shows the distinct k-mers shared between the assembled haplotypes and the reads (green), the distinct k-mers shared between the reads and each respective haplotype (red and blue), along with the distinct k-mers only found in the reads (black).

**
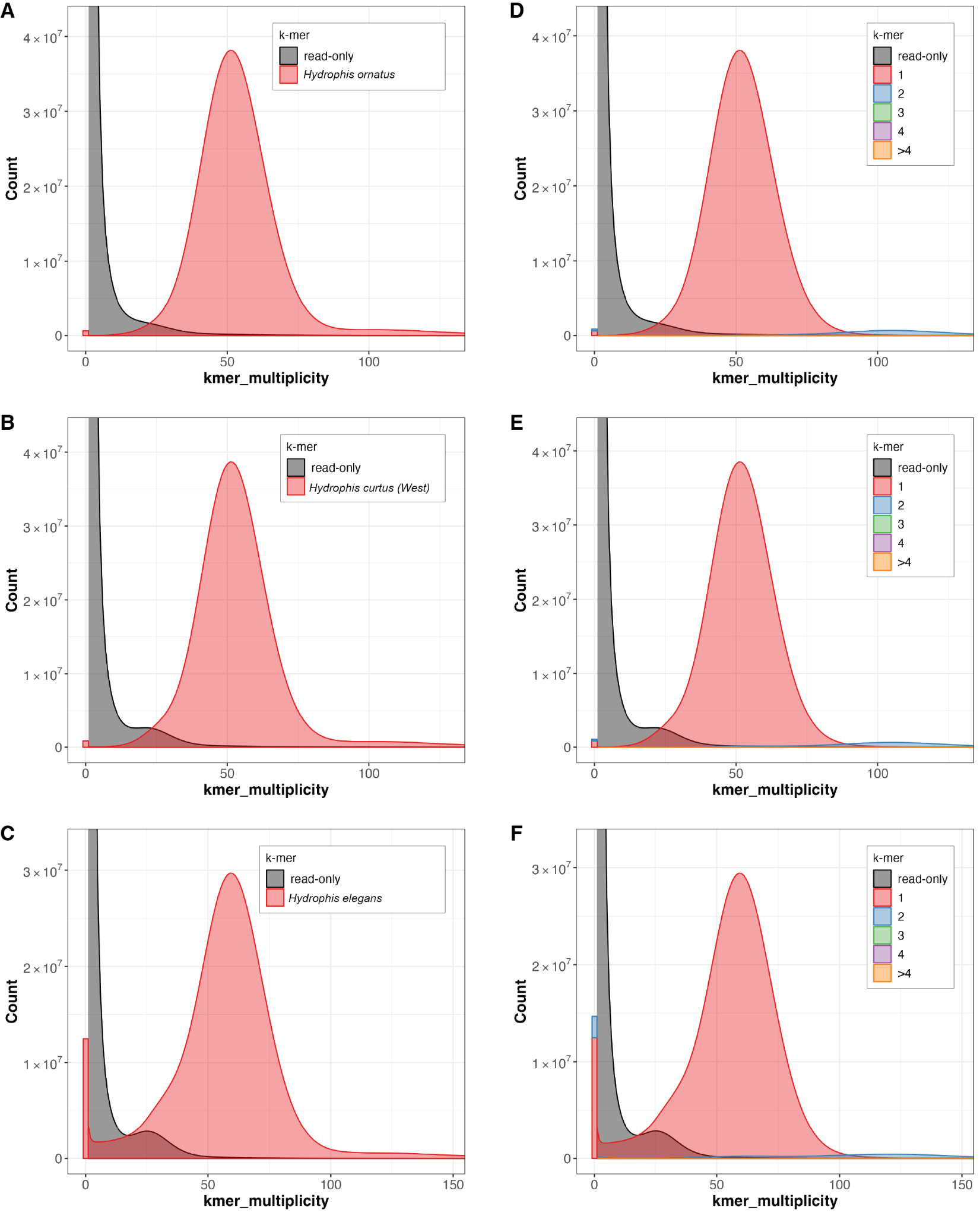
**

**Fig. S7:** Merqury k-mer spectra analysis for Nanopore assembled snakes*.* A-C) Assembly spectra plots showing the distinct k-mers shared between each assembly (*H. ornatus, H. curtus (West)* and *H. elegans* respectively*)* and their reads (red). k-mers only found in the read-set are shown in black. D-F) Spectra plots showing the k-mer multiplicity for the distinct k-mers in each respective genome. k-mers appearing once in each genome are coloured red, twice in blue, three times are green and four or more times in purple and orange respectively. K-mers that are not shared between the reads and assembly are shown in black.

**
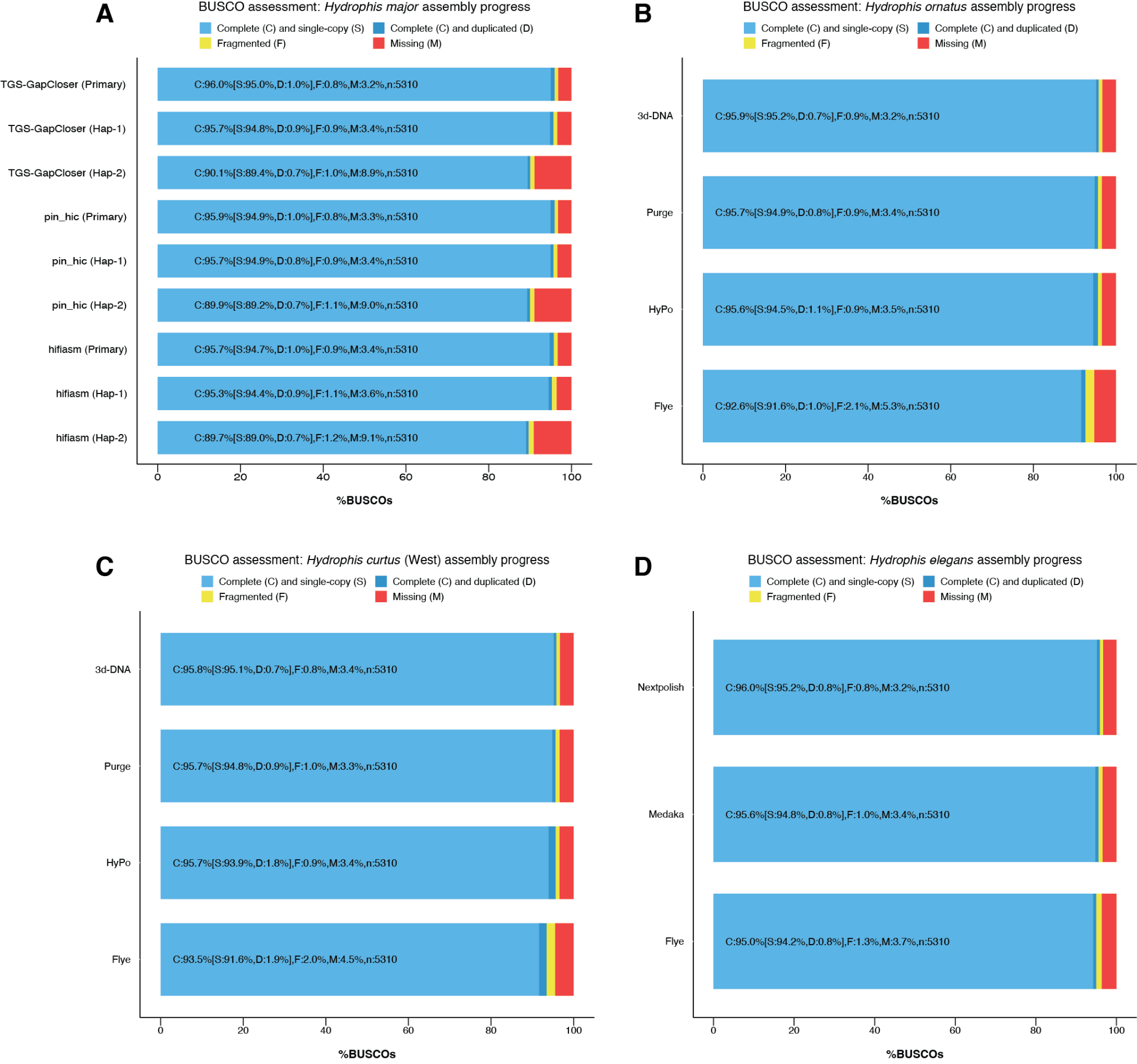
**

**Fig. S8:** BUSCO completeness during assembly. Assembly stage is presented along the y-axis, and percentage of BUSCO’s is along the x-axis. A) BUSCO was run on the primary and dual assemblies of *Hydrophis major* at each stage of the assembly pipeline from the initial contigs (*hifiasm*) to the final gap-closed assembly (TGS-GapCloser). B-C) BUSCO scores for each assembly stage of the *H. ornatus* and *H. curtus* (West) assemblies. Flye represents the initial contig assemblies, with 3d-DNA representing the final chromosomes. D) The assembly progression for *H. elegans*, in which Nextploish represents the final assembly, with Flye and Medaka representing the initial assembly and Nanopore-polished stages respectively.


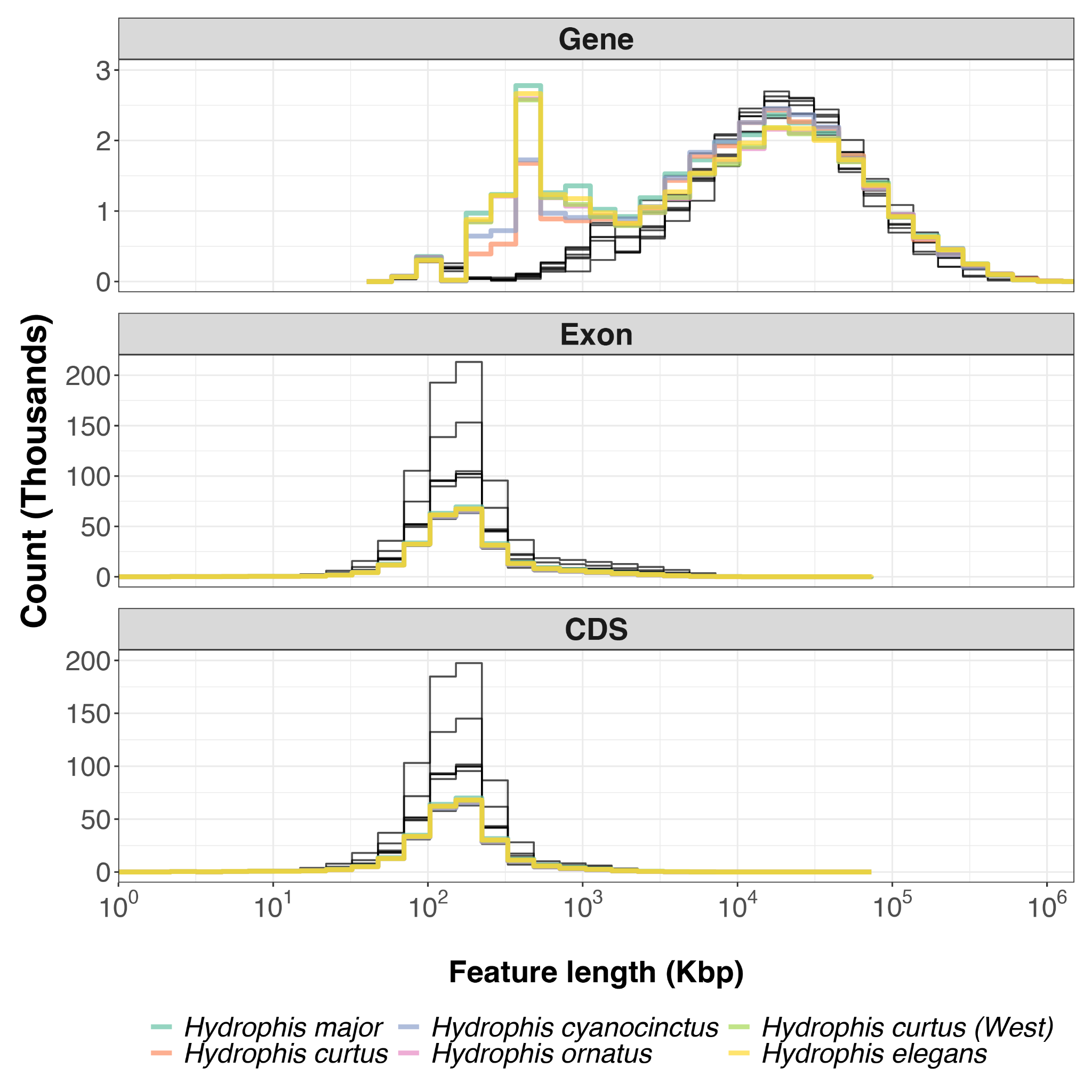


**Fig. S9:** Length distribution of gene features. Gene, exon and coding-sequence (CDS) lengths (x-axis) are plotted against their total count (y-axis) for each of the six newly assembled *Hydrophis* snakes (coloured lines). The black lines represent gene annotations for RefSeq annotated snakes including *Notechis scutatus*, *Pseudonaja textilis*, *Thamnophis elegans*, *Pantherophis guttatus*, *Protobothrops mucrosquamatus*, *Crotalus tigris* and *Python bivittatus*. Each of the features show similar length profiles, however there is an excess of shorter gene models only found in the *de novo* gene annotations that aren’t present in the RefSeq annotated snakes.


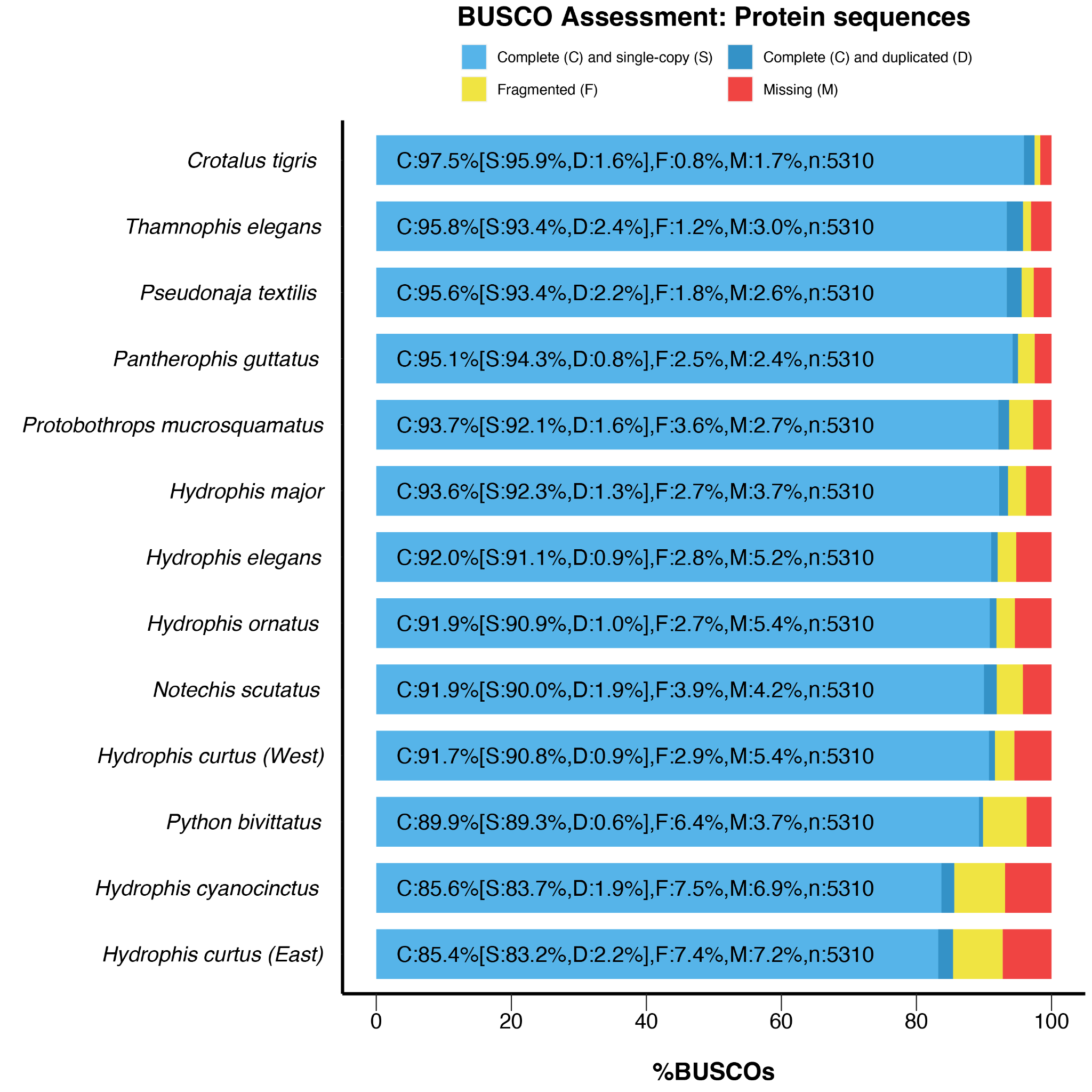


**Fig. S10:** BUSCO completeness of longest-isoform protein sequences. The longest isoform of each gene was extracted using AGAT, before running BUSCO on the resulting peptide files.


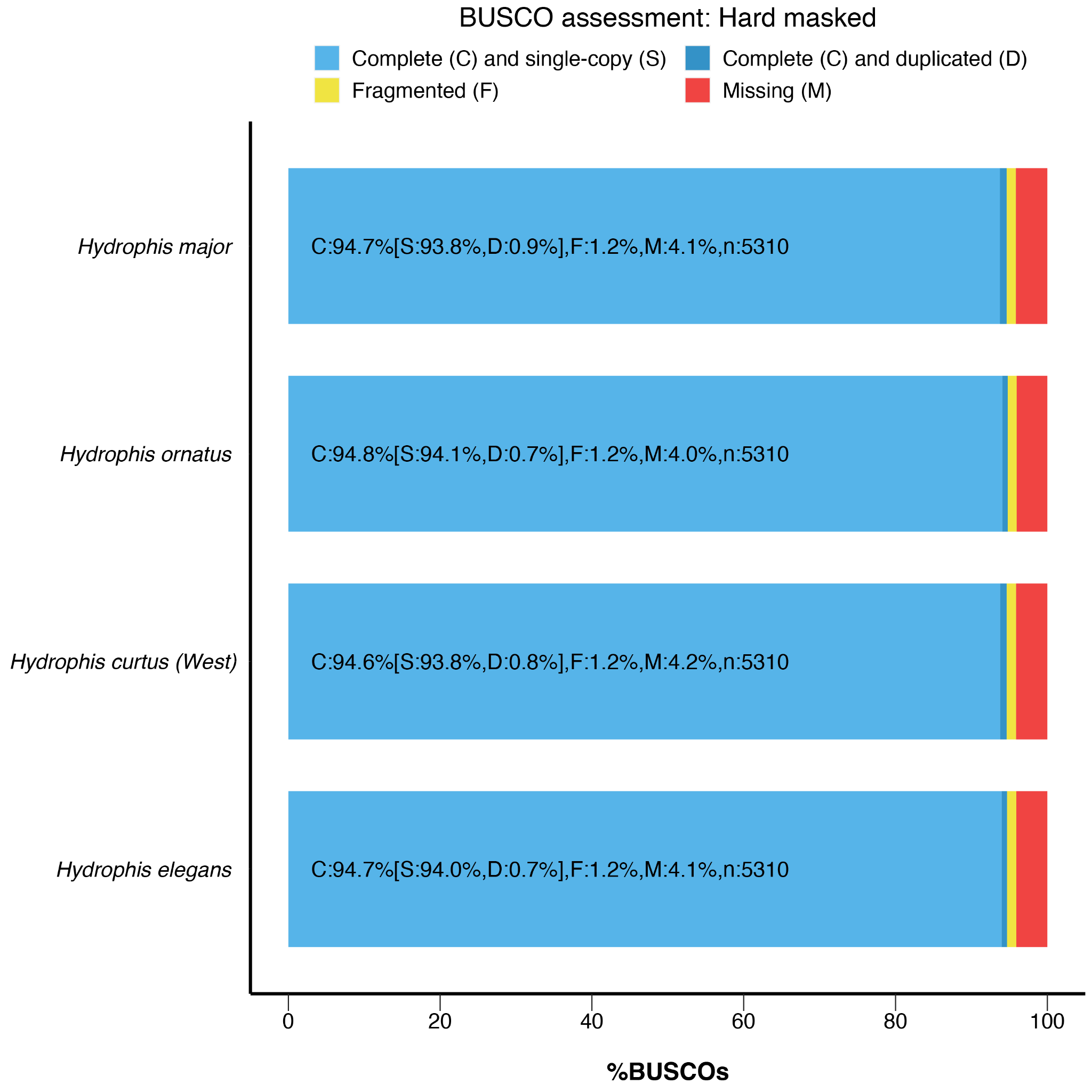


**Fig. S11:** BUSCO completeness post hard-masking. Each genome had annotated repeat elements hard-masked (replaced with N’s), before running *BUSCO* using the tetrapoda_odb10 database. Hard-masking the genomes has little effect on the gene content, dropping the complete *BUSCO* measure by an average of 1.3% across all four snakes, indicating the annotated repeats constitute repetitive elements rather than non-repetitive genetic sequence.

**
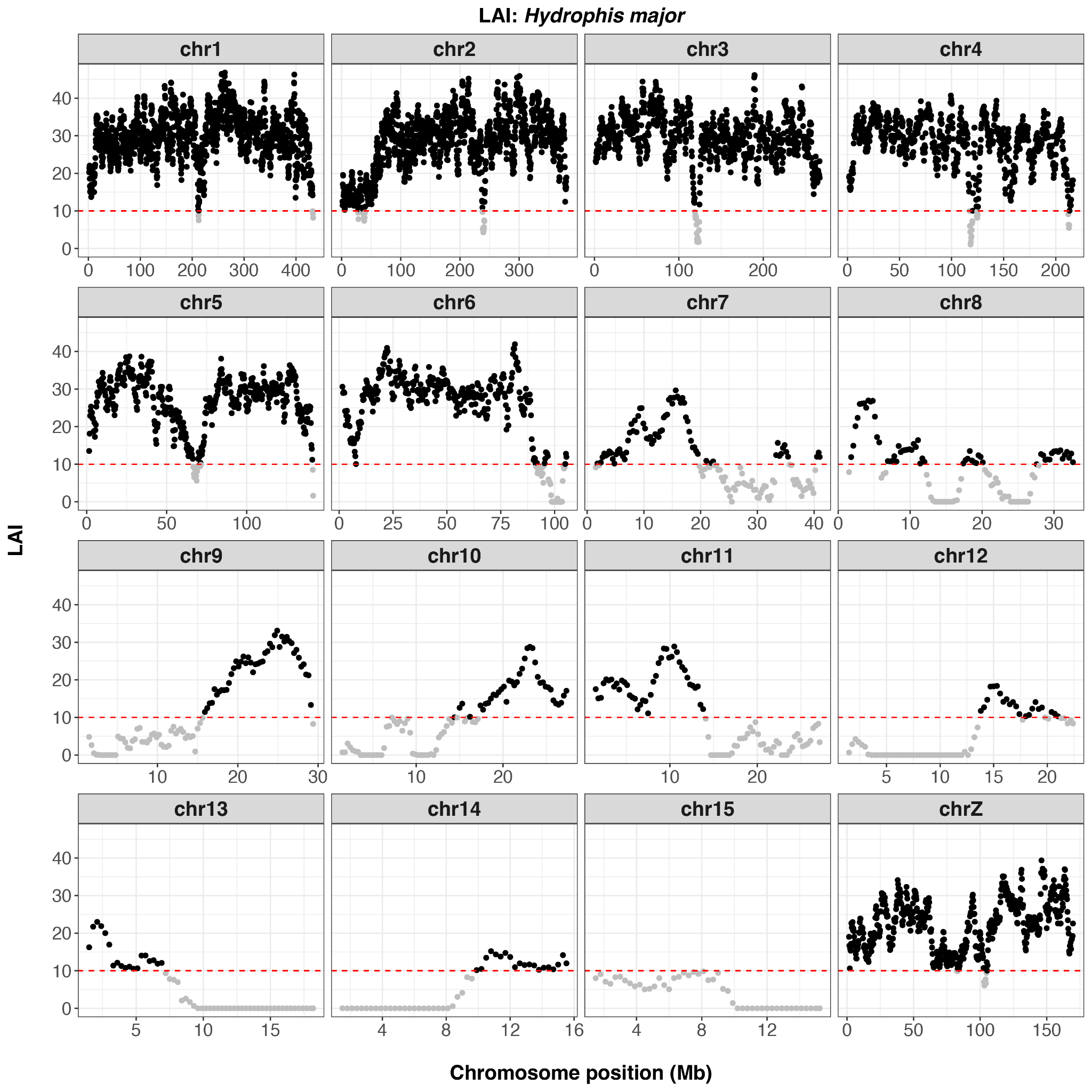
**

**Fig. S12:** LTR Assembly Index (LAI) scores for *Hydrophis major* chromosomal sequences. LAI scores were calculated in 3Mbp windows with a 300Kbp step-size. The red dashed line at y=10 indicates the ‘reference quality’ cutoff.


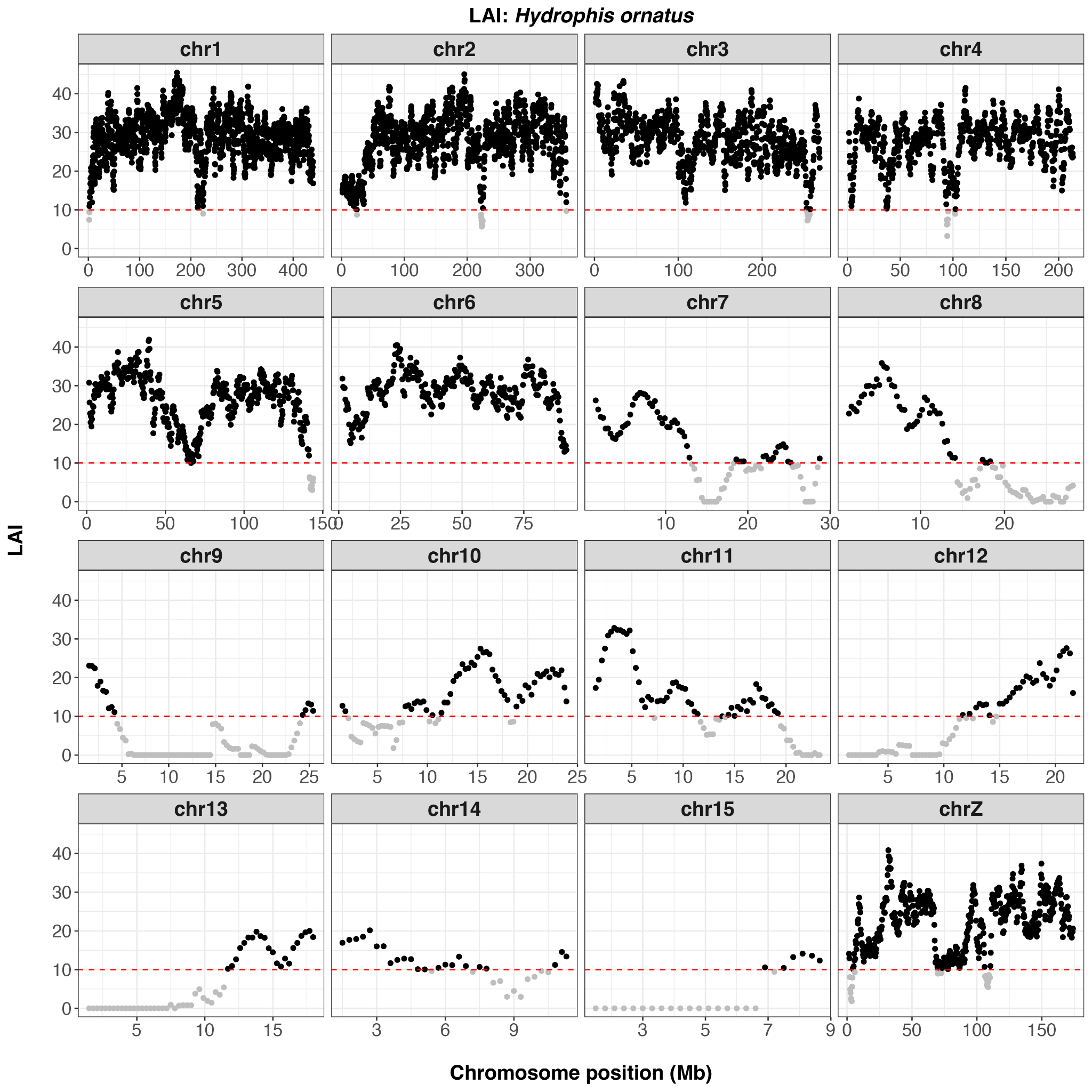


**Fig. S13:** LTR Assembly Index (LAI) scores for *Hydrophis ornatus* chromosomal sequences. LAI scores were calculated in 3Mbp windows with a 300Kbp step-size. The red dashed line at y=10 indicates the ‘reference quality’ cutoff.


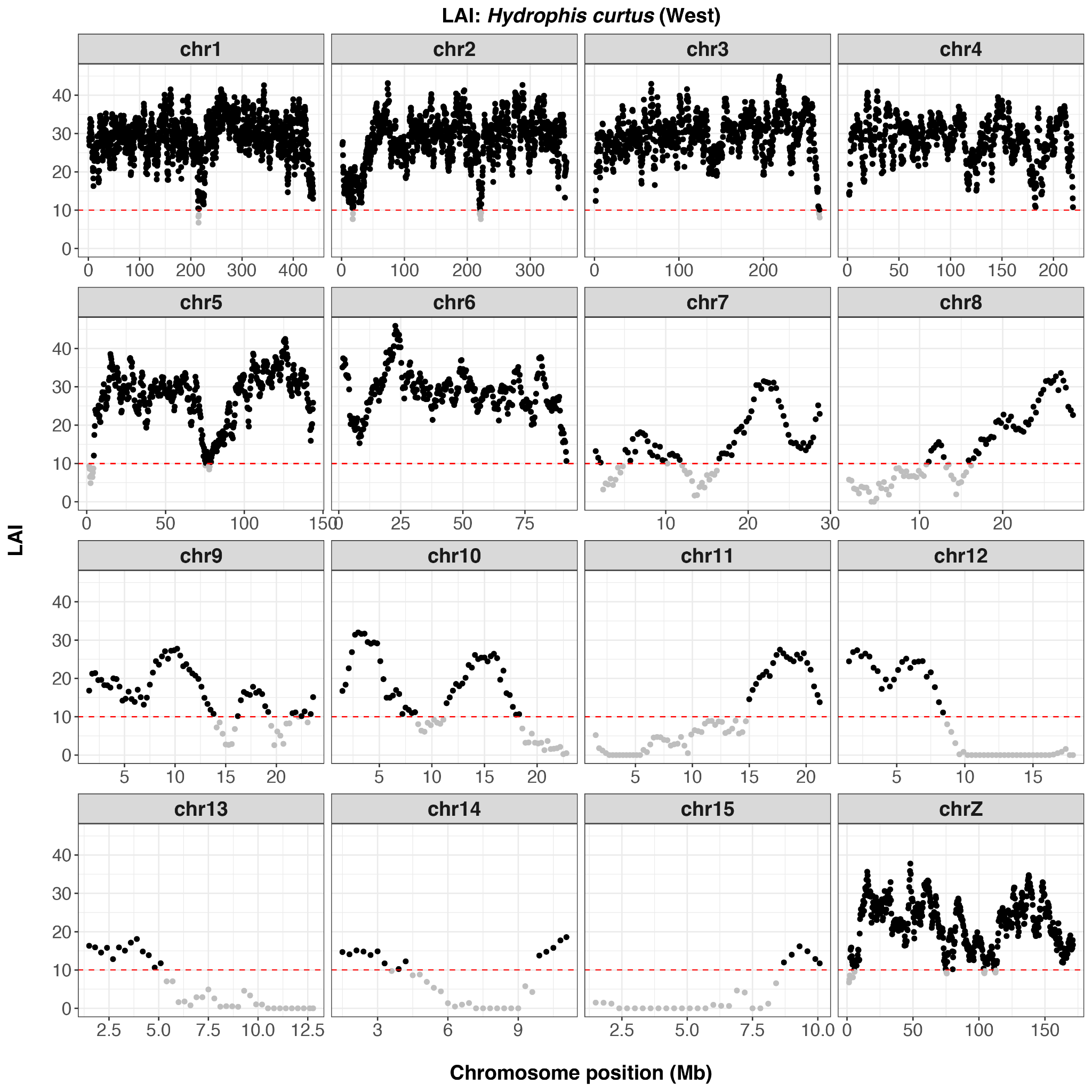


**Fig. S14:** LTR Assembly Index (LAI) scores for *Hydrophis curtus* (West) chromosomal sequences. LAI scores were calculated in 3Mbp windows with a 300Kbp step-size. The red dashed line at y=10 indicates the ‘reference quality’ cutoff.


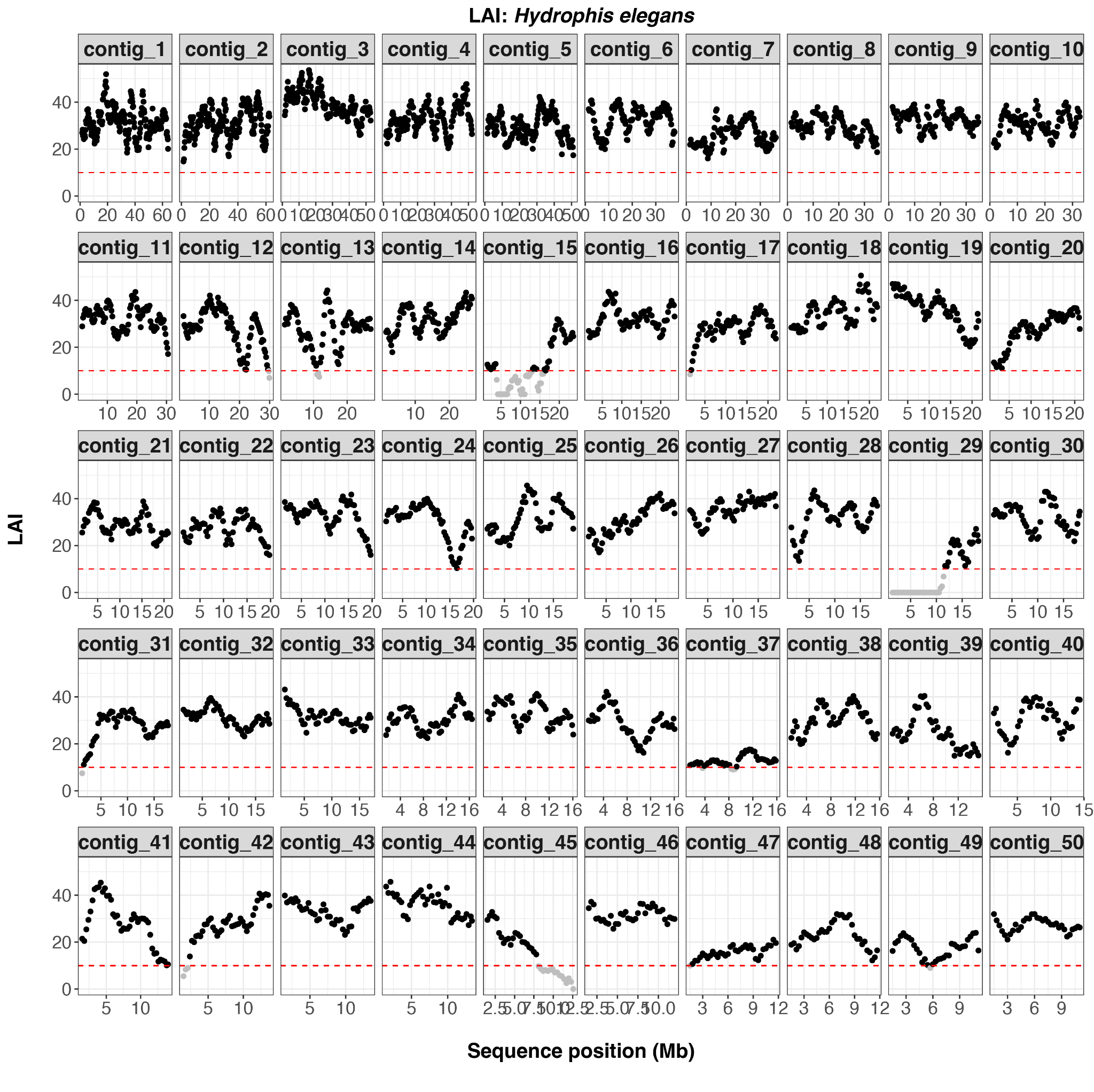


**Fig. S15:** LTR Assembly Index (LAI) scores for the fifty largest *Hydrophis elegans* contig sequences. LAI scores were calculated in 3Mbp windows with a 300Kbp step-size. The red dashed line at y=10 indicates the ‘reference quality’ cutoff.


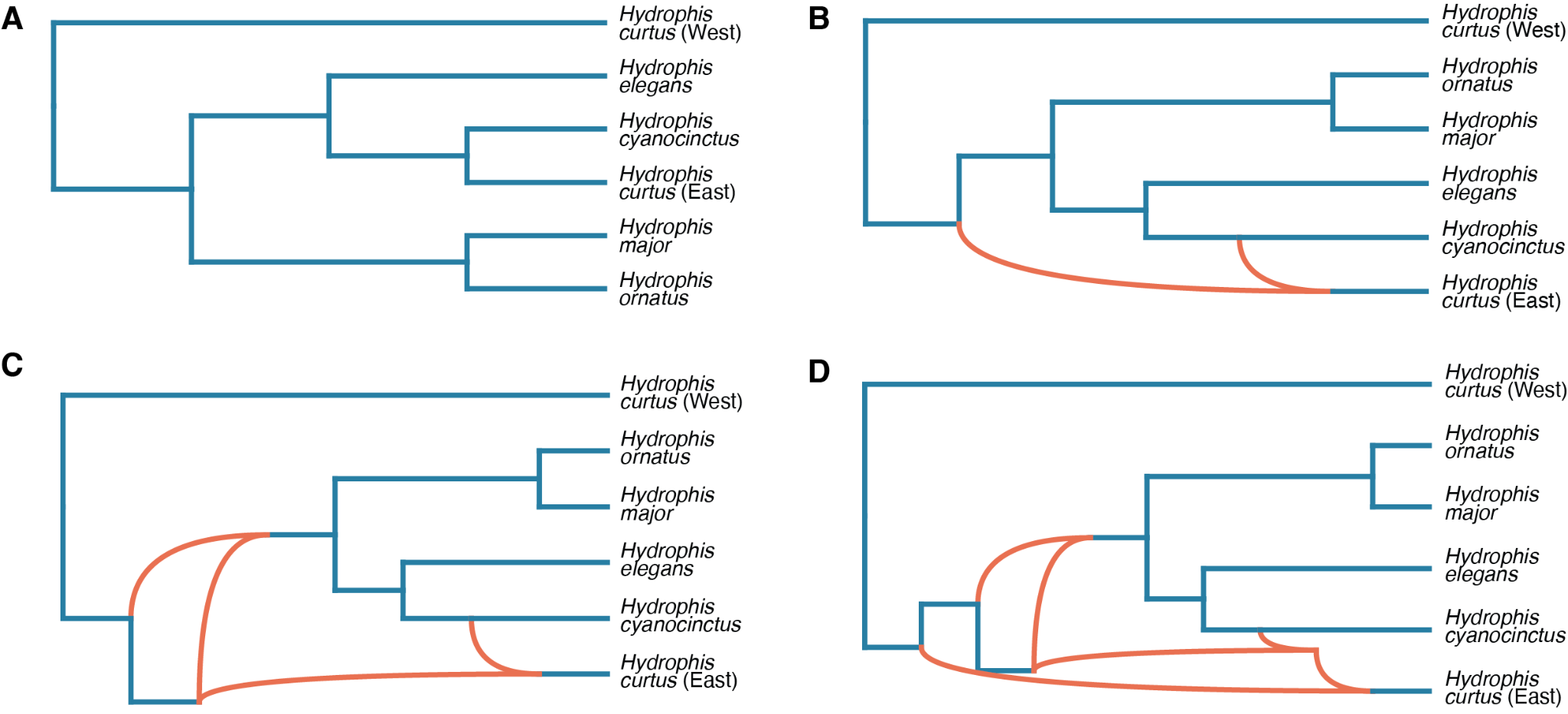


**Fig. S16:** PhyloNet networks for varying reticulation values. Multiple *PhyloNet* networks were produced by adjusting the number of reticulation events that were allowed to occur. Facets A-D represent zero through three reticulation events, respectively.


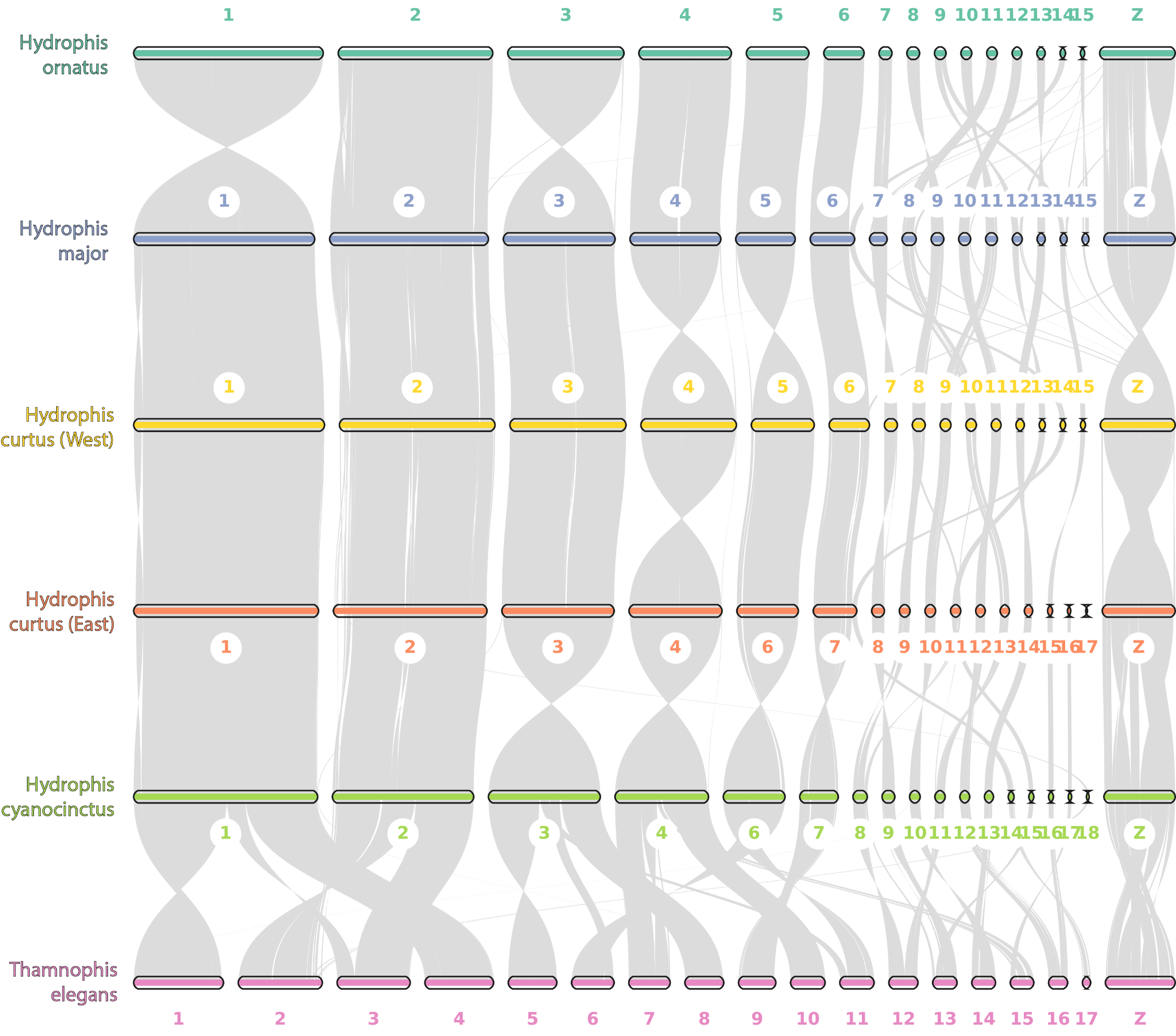


**Fig. S17:** MCscan synteny before reverse complementing select sequences and arranging chromosomes in order. Some examples of fully inverted chromosomes include chromosomes 1 and 3 between *H. ornatus* and *H. major*, chromosomes 3, 4, 6 and 7 between *H. curtus (East)* and *H. cyanocinctus* and chromosome 4 between *H. curtus (West)* and *H. curtus (East)*.


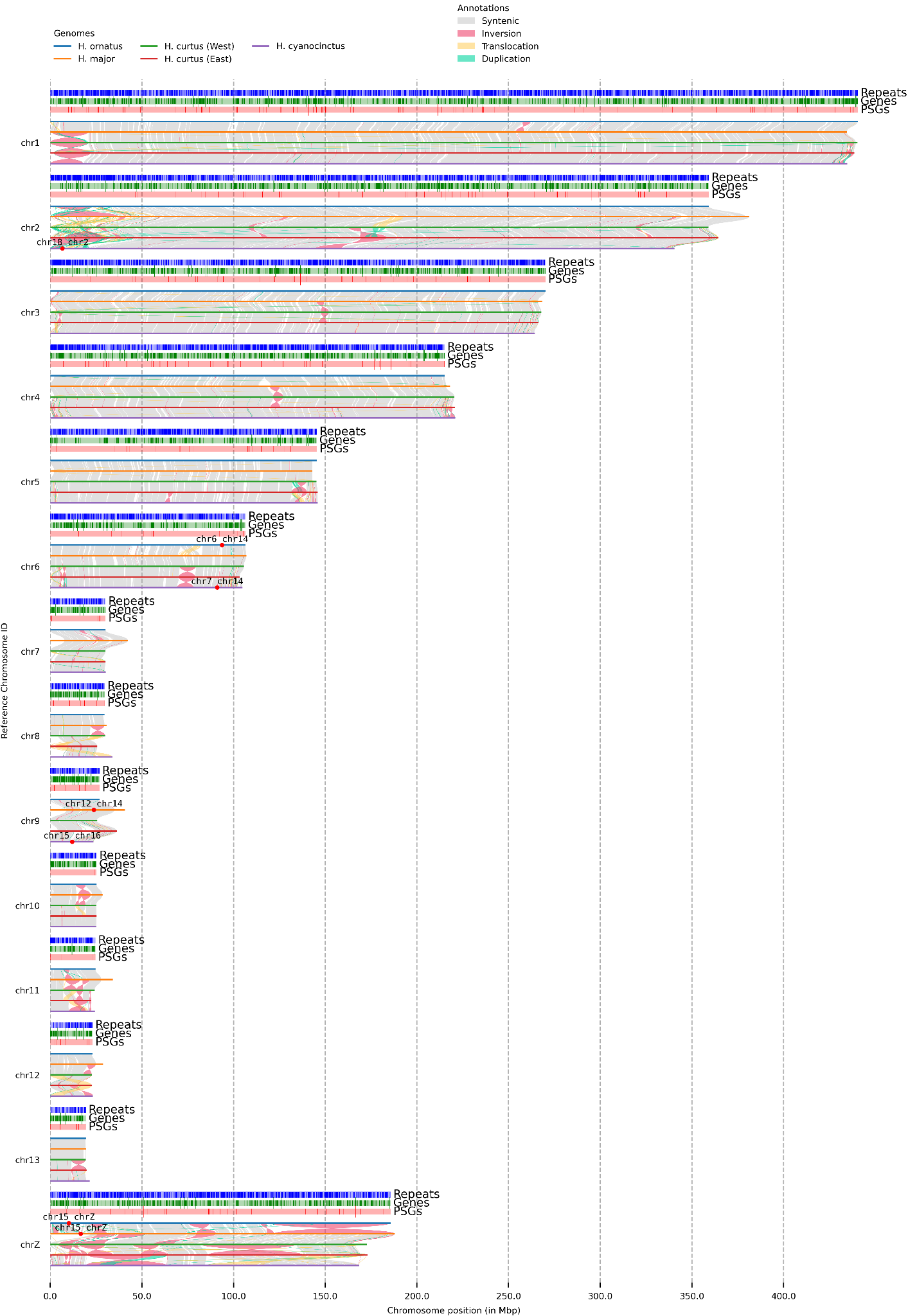


**Fig. S18:** Chromosome synteny and structural variation between five *Hydrophis* snakes. Chromosome synteny and structural rearrangements were visualised using Syri and Plotsr from pairwise whole genome alignments between the *Hydrophis* snakes. Each snake in the figure is compared to the genome below it, with *Hydrophis* *ornatus* being used as the reference anchor, with all annotation tracks relative to it. Chromosome sequences were modified in each of the snakes so that their karyotypes were identical. Chromosomes that were concatenated together are labeled in the figure for each respective snake. Structural variation is coloured with inversions in red, translocations in yellow and duplications in green.

**Figure S19:** Venn diagram of marine-specific PSGs identified by PAML (drop-out) and BUSTED-PH.
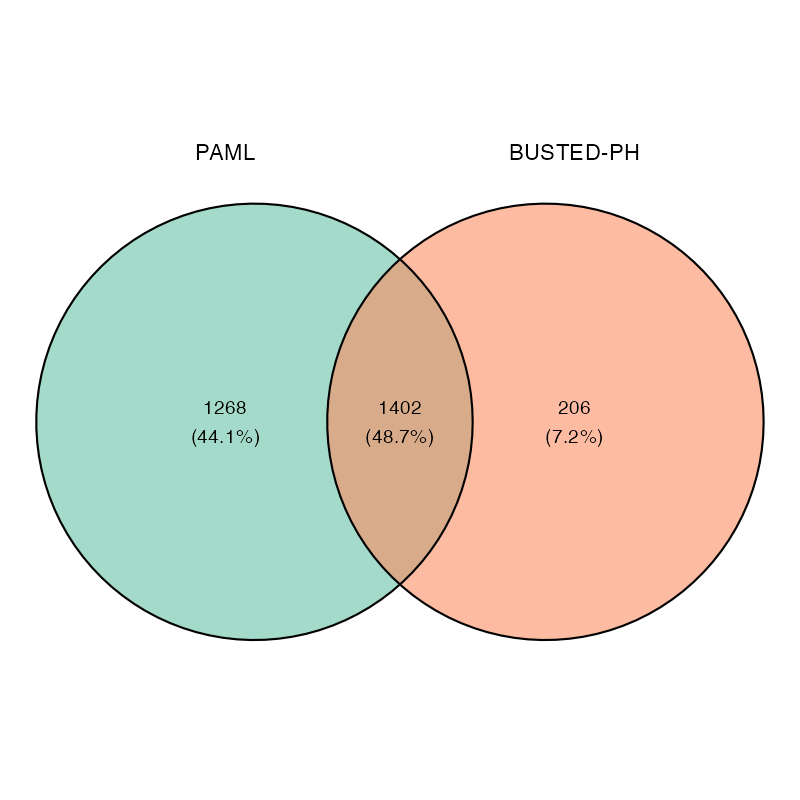


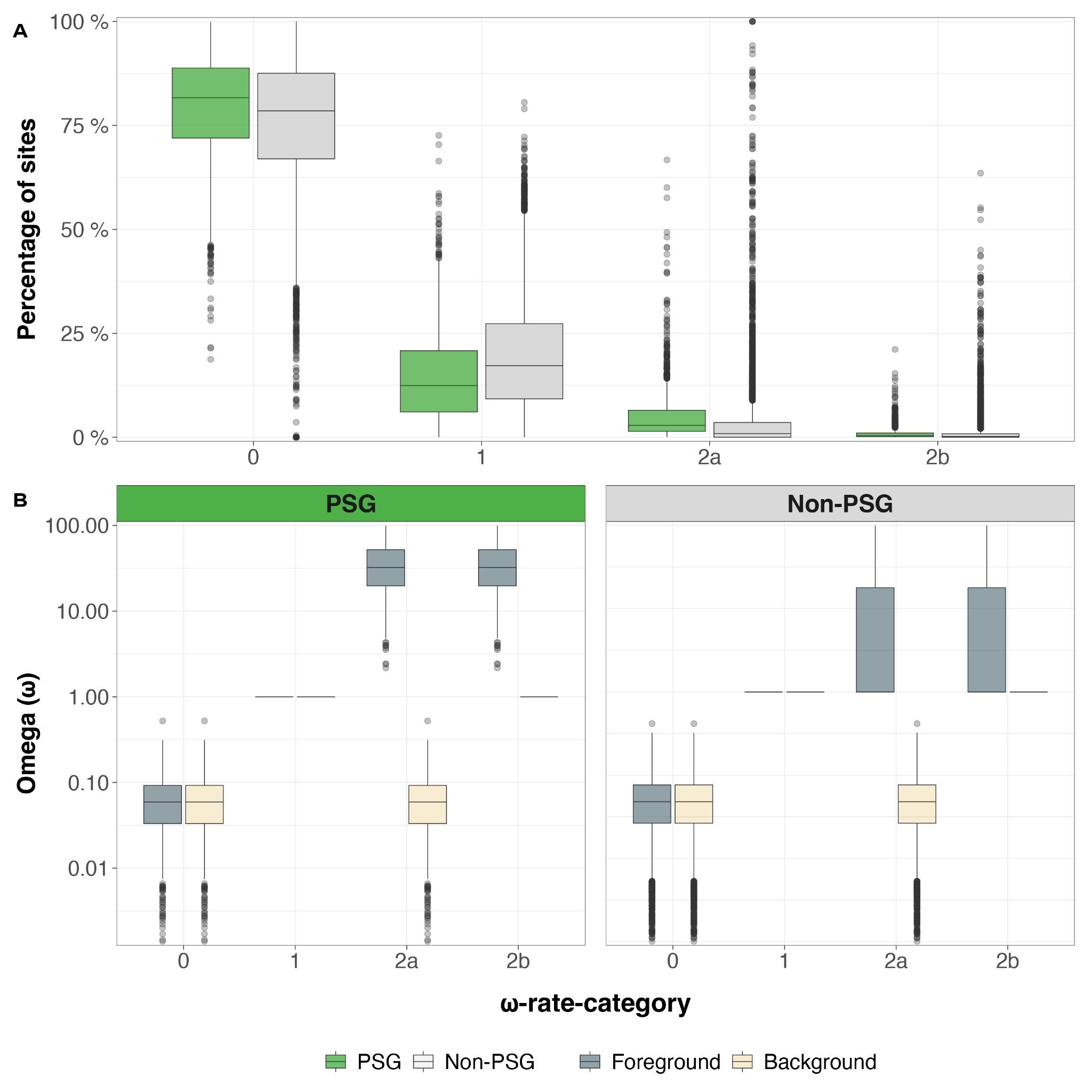


**Fig. S20:** PAML Branch-Site (alt.) summary. A) Percentage of sites in each multiple sequence alignment belonging to each *ω-*rate class. Green boxes represent *Hydrophis* positively selected genes with grey representing terrestrial snakes. Classes 0 and 1 represent instances where positive selection is prohibited (*ω < 1* and *ω = 1*, respectively), while 2a and 2b represent situations where *ω > 1* is allowed in the foreground branches, but not in the background branches. B) Comparison of *ω* values between PSG and non-PSG genes for each rate-category. PSGs have considerably higher *ω* values in 2a and 2b relative to the background branches, while non-PSGs show very low *ω* values for the foreground snakes relative to the background snakes, which show higher *ω* values in 2a and 2b.


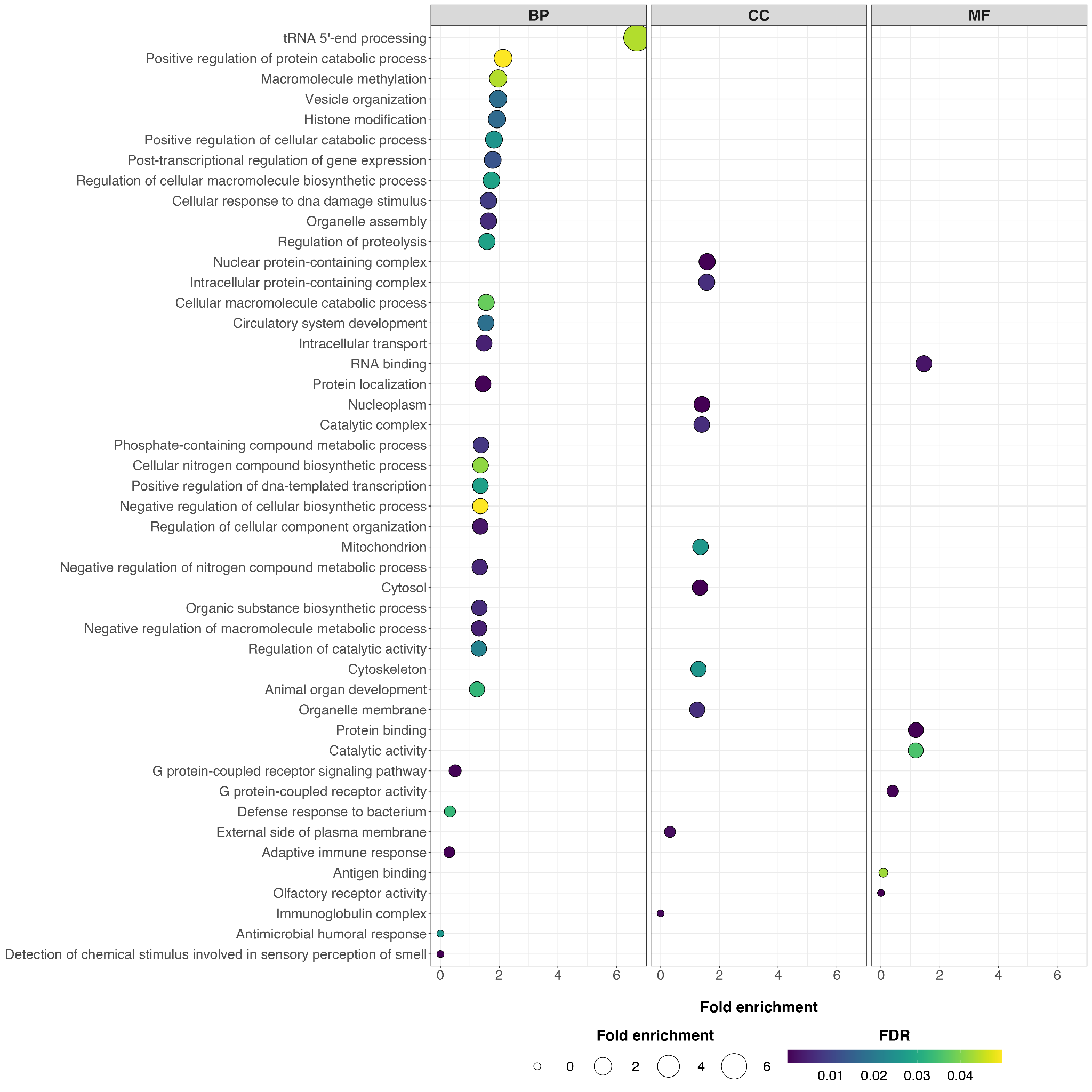


**Fig. S21:** PANTHER fold enrichment for the most specific over-represented terms (level 0). Fold enrichment represents the observed number of genes associated with a GO term divided by the expected number of genes for the term. Values greater than 1 indicate overrepresentation while values less than 1 represent underrepresentation. Facets represent the three ontologies with the size of the circles representing the fold enrichment, and the colour indicating the FDR value.
